# Senolytic effects of a modified Gingerenone A

**DOI:** 10.1038/s41514-025-00230-3

**Published:** 2025-05-30

**Authors:** Ruin Moaddel, Chad Sanehira, Gregory Keyes, Chang-Yi Cui, Reza Ahmadkhaniha, Julián Candia, Nathan L. Price, Sarah Eckroth, Bryce Middleton, Mohammed Khadeer, Caio H. Mazucanti, Ross A. McDevitt, Myriam Gorospe, Rafael de Cabo, Josephine M. Egan, Christopher E. Ramsden, Luigi Ferrucci

**Affiliations:** 1 Laboratory of Clinical Investigation, National Institute on Aging, Intramural Research Program, NIH, Baltimore, MD USA; 2 Translational Gerontology Branch, National Institute on Aging, Intramural Research Program, NIH, Baltimore, MD USA; 3 Laboratory of Genetics and Genomics, National Institute on Aging, Intramural Research Program, NIH, Baltimore, MD USA; 4 Comparative Medicine Section, National Institute on Aging, Intramural Research Program, NIH, Baltimore, MD USA

**Keywords:** Chemical biology, Drug discovery, Therapeutics

## Abstract

Senescent cells accumulate with aging and are associated with several age-associated diseases and functional declines. Eliminating senescent cells with senolytics improves aging phenotypes in mouse models and may improve the health of people with chronic diseases. To date, very few senotherapeutic (senolytics and senomorphics) compounds have been identified. In a recent study, we reported that gingerenone A (GinA) has a senolytic effect via mechanisms including the activation of caspase-3 activity and apoptotic cell death. In this study, we investigated whether GinA has senotherapeutic properties in a mouse model of senescence. Moreover, we modified GinA with eicosapentaenoic acid (EPA) esters (GinA-EPA) or docosahexaenoic acid (DHA) esters (GinA-DHA) to generate modified gingerenone A (modGinA) that could enhance GinA effects. We found that both GinA and modGinA induced biochemical and histological changes consistent with anti-inflammatory, senolytic, and senomorphic effects, leading to improved metabolic and mitochondrial functions.

## Introduction

Senescent cells exhibit irreversible replication arrest, as first described by Hayflick^1^, at least in part due to increased expression of the growth inhibitory proteins TP53 (p53), CDKN1A (p21) and CDKN2A (p16)^2^. Senescent cells secrete a large number of biologically active molecules globally termed the senescence-associated secretory phenotype (SASP), which include pro-inflammatory cytokines, growth factors, matrix metalloproteases and signaling lipids^3^. It has been proposed that senescence is beneficial at younger ages, as it plays a role in wound healing, tissue remodeling, embryonic development, and tumor suppression^4^, although evidence for these functions is still scant in humans^5^. However, it is widely recognized that accumulation of senescent cells in older ages is detrimental and contributes to chronic inflammation, liver and lung fibrosis, atherosclerosis, insulin resistance, neurodegenerative disorders, and cancer^6^. In animal models, elimination of senescent cells by senolytic compounds or by genetic approaches improves health span and slows down the development of aging phenotypes that are associated with functional impairment^7,8^. Whether the clearance of senescent cells in humans can effectively prevent or cure age-related chronic diseases and functional impairments is currently being investigated in many randomized controlled trials^7,9,10^.

Senotherapeutics include senolytic compounds that selectively eliminate senescent cells by poorly understood mechanisms including the unleashing of apoptosis, and senomorphic compounds that can modulate the SASP, reducing harmful effects without removing the cells. To-date, effective senotherapeutic compounds include navitoclax^11^, fisetin and piperlongumine^12^. The senolytic cocktail of dasatinib and quercetin (D + Q)^13^ appear to clear senescent cells both in culture and in organisms. We have previously shown that the total extract from *Zingiber officinale Rosc*. (ginger) reduced both senescent cell viability and senescence-associated-β-galactosidase (SA-β-Gal) activity^14^. Using a candidate approach we found that Gingerenone A (GinA) accounted for most of the effects of ginger on senescent cells and therefore should be considered a novel senolytic compound^14^. GinA activates caspase-3 in senescent cells, suggesting that one of its mechanisms of action is to enhance the vulnerability of senescent cells to apoptosis. In addition, GinA has senomorphic activity, reflected in its ability to diminish the secretion of the SASP proinflammatory cytokines IL-6, CCL2 and IP-10, and to increase the levels of anti-inflammatory IL-13 from irradiated fibroblasts^14^. In a separate study, GinA was found to prevent local inflammation by inhibiting macrophage recruitment and regulating inflammatory cytokine expression^15^.

**Fig. 1:**
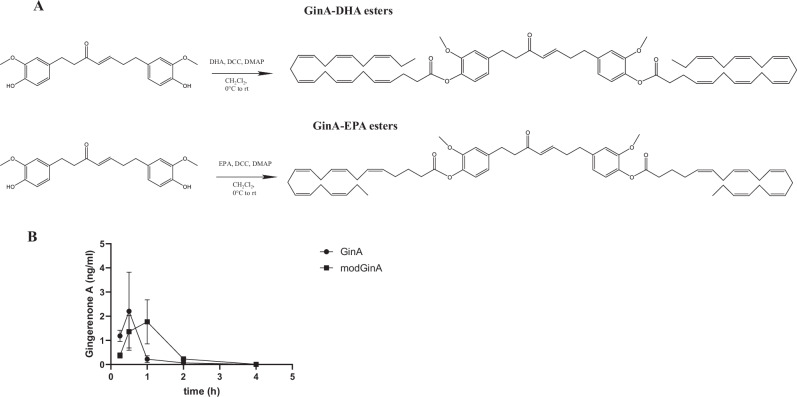
Synthesis and distribution of modified Gingerenone A (modGinA). **A** Synthesis of GinA-DHA esters and Gin-EPA esters. modGinA is an equimolar mixture of these esters. **B** Circulating levels of gingerenone A (GinA) following oral administration of 10 mg/kg of GinA (*n* = 4) or 27 mg/kg modGinA (*n* = 4) to 19 month-old C57BL/6JN mice. Averages with standard error are reported.

Here, we aimed to enhance the therapeutic anti-inflammatory effects of GinA by modifying it with either two eicosapentaenoic acid (EPA) esters (GinA-EPA) or two docosahexaenoic acid (DHA) esters (GinA-DHA) (Fig. 1A). EPA and DHA supplementation is known to decrease circulating proinflammatory cytokines^16^, and in patients undergoing kidney transplantation, supplementation with omega-3 polyunsaturated fatty acid, EPA and DHA, decreased the presence of SASP in plasma^17^. We conducted a comparative study of orally administered GinA versus an equimolar mixture of GinA-DHA and GinA-EPA (modGinA) versus placebo in aged mice to determine whether both treatment groups display anti-inflammatory and/or senolytic activity in vivo and whether modified forms of GinA have more pronounced anti-inflammatory and/or senolytic effects than unmodified GinA.

## Results

### Circulating levels of GinA after administration of GinA and an equivalent concentration of modified GinA

Molar equivalents of GinA (10 mg/kg) or modGinA (an equimolar mixture of GinA-DHA and GinA-EPA; 27 mg/kg) were administered by oral gavage and serum was collected at multiple timepoints after administration (0.25, 0.5, 1, 2 and 4 h) (Fig. 1B). Although not statistically significant, peak serum concentration (C_max_) of GinA was higher following GinA (2.54 ± 1.94 ng/ml at 0.5 h) as compared to modGinA administration (1.77 ± 0.91 ng/ml at 1 hour), whereas GinA total systemic exposure (Area Under the Curve (AUC_0−t_)) was higher following modGinA (2.24 ± 1.11 h·ng/ml) compared to GinA administration (1.25 ± 1.13 h·ng/ml).

**Fig. 2:**
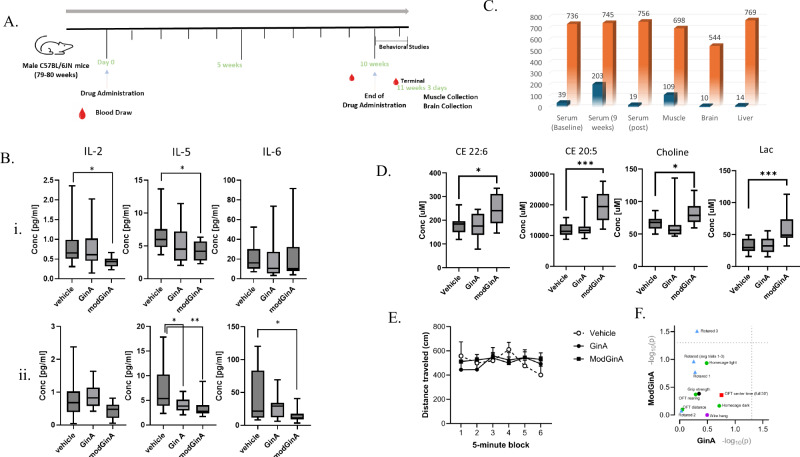
Chronic administration of GinA and modGinA in aged C57BL/6JN mice. **A** Study procedures for daily oral administration of GinA (10 mg/kg, *n* = 12) or modGinA (27 mg/kg, *n* = 14) or vehicle (high oleic acid sunflower oil, *n* = 12) in ~80 week old C57BL/6JN mice for 10 weeks. Blood was collected at 9 weeks during treatment and 10 days after termination of treatment, with brain, muscle and liver also collected. Behavioral studies were carried out following termination of treatment over a period of 10 days. **B** Circulating levels of IL-2, IL-5 and IL-6 during treatment (i) and after treatment (ii). Proteins level significantly different between treatment groups and vehicle are labeled (**p < 0.01; *0.01 < *p* < 0.05, Dunnetts test). **C** Total number of metabolites above the limit of detection (orange) in >70% of samples and total number of significant metabolites (blue) identified in serum, muscle, brain and liver using the unadjusted Kruskal–Wallis test. **D** Circulating levels of CE 22:6, CE 20:5 and choline in serum during treatment (9 weeks). Data were calculated using the unadjusted Kruskal–Wallis test. **E** Locomotor activity in an open field chamber was unaffected by treatment throughout the full 30 min of testing **F** Scatter-plot for all the behavioral tests carried out after termination of treatment and within a 10-day window post-treatment.

GinA (10 mg/kg/day), modGinA (27 mg/kg/day) or vehicle (high oleic sunflower oil) were administered daily by oral gavage for 10 weeks to C57BL/6JN mice (79–80 weeks old) (Fig. 2A). Blood (for serum) was collected prior to drug treatment (baseline) and after the 9th week of treatment. Additionally, behavior tests were performed (see Methods), followed by collection of post-mortem blood as well as liver, muscle and brain tissue 10 days after the last oral dose (washout). During the course of the study, three mice died: two in the GinA and one in the vehicle group. These mice were censored from all analyses. None died in the modGinA group.

### Serum inflammatory markers

Circulating levels of cytokines/chemokines (Table 1) were quantified in serum at multiple timepoints and in muscle, liver and brain only after washout. At baseline there were no significant differences between treatment groups and vehicle with respect to inflammatory markers (Table 1 and S1A). After the 9^th^ week of treatment, IFN-γ, IL-10 and IL-1β levels were significantly different between groups (vehicle, GinA and modGinA) by one-way ANOVA (Table 1). Compared to vehicle, GinA treatment was associated with increased levels of IL-10 and TNFα, while modGinA was associated with reduced IL-2 and -5 (Table 1). At washout, IL-2, -5 and -6 were significantly different between groups by one-way ANOVA (Table 1; Fig. 2b). Compared to vehicle, modGinA was associated with reduced IL-5 and -6. IL-6 is a key secreted SASP factor, IL-2 stimulates both effector immune cells and regulatory T (Treg) cells^18^ and IL-5 is central to the initiation and sustenance of eosinophilic airway inflammation^19^. In muscle, liver and brain none of the inflammatory markers evaluated were significantly different between groups (Table S1B).

**Table 1:**
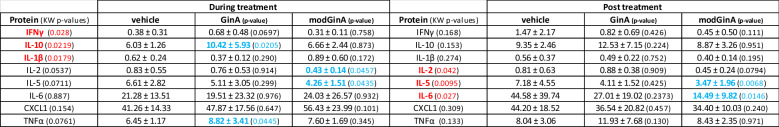
Circulating cytokine levels (pg/ml) in mice treated with 10 mg/kg GinA or 27 mg/kg modGinA daily for 10 weeks

Blood was collected at 9 weeks (During treatment) or 10 days after treatment washout (Post treatment). Proteins that were significantly different by non-parametric one-way anova analysis (Kruskal-Wallis test) are in red font (p-values are in parenthesis). Concentrations in blue font were significant in follow-up (Dunnetts test comparing treatment groups to vehicle).

### Metabolomics in serum during treatment

Thirty-nine, 174, and 11 metabolites were significantly different between all three groups at baseline, during treatment and after washout, respectively (Table S2). Of the 39 metabolites that were different between groups at baseline, only 7 (indole sulfate, phosphatidylcholines [PC] 40:2, O-32:2, O-38:3, as well as phosphatidylglycerol [PG] 16:0_20:3 hexosyl-ceramides [Hex-Cer] d18:2/24:0 and sphingomyelin [SM] 42:1) were significantly altered by treatment.

Longitudinal analysis was carried out to identify treatment-dependent (between vehicle, GinA, modGinA) changes in metabolites (Table 2; Table S3). The metabolites that changed included threonine, GABA, β-alanine, anserine, L-alpha-aminoadipic acid (alpha-AAA), lactate, choline and p-cresol sulfate. The most evident changes involved lipid substrates; cholesterol esters (CE), ceramides (Cer), SM, lysolipids, glycerides, and phospholipids (PL) (Table 2; Table S3). Of interest, three of the top five most significant changes in the longitudinal analysis contained EPA (20:5) (phosphatidyl ethanolamine [PE] P-16:0/20:5, 20:0/20:5 and 18:0/20:5), with all three increasing after the 9th week of modGinA treatment (Table 3; Table S2), consistent with modGinA containing DHA and EPA.

**Table 2:** Metabolites that changed in the longitudinal analysis from serum collected at baseline, 9 weeks and after washout in vehicle, GinA (10 mg/kg) and modGinA (27 mg/kg)

Class	Metabolites
Amino Acids, amino acid related and Biogenic Amines	alpha-AAA,Anserine,beta-Ala,Choline,GABA, Thr
Cholesterol Esters (10)	CE 14:0,CE 15:0,CE 16:0,CE 16:1,CE 18:1,CE 18:2,CE 18:3,CE 20:3,CE 20:4,CE 20:5
Ceramides (5)	Cer d18:1/24:0,Cer d18:1/24:1,Cer d18:2/24:0, Hex-Cer d18:1/20:0,Hex-Cer d18:1/24:1
Sphingomyelins (7)	SM 34:1,SM 35:1,SM 36:1,SM 36:2,SM 42:1,SM 42:2,SM 43:1
Carboxylic acids	Lactic Acid
Cresol	p-Cresol-SO4
Lysolipids (2)	LPE 18:0,LPE P-18:1
Glycerides (3)	MG 18:1,MG 20:3, TG 18:2_36:0
Phosphatidic Acids (11)	PA 16:2_18:1,PA 17:1_18:1,PA 17:2_18:1,PA 18:0_18:1,PA 18:1_18:1,PA 18:1_18:3,PA 18:1_18:4,PA 18:1_20:3,PA 18:1_22:3,PA 18:2_18:2,PA 18:2_22:3
Phosphatidylcholines (2)	PC O-34:0,PC O-36:0
Phoshpatidylethanolamines (11)	PE 33:1,PE P-16:0/15:0,PE P-16:0/16:0,PE P-16:0/18:1,PE P-16:0/20:5,PE P-18:0/18:1,PE P-18:0/20:5,PE P-18:1/18:1,PE P-18:1/18:2,PE P-18:1/20:4,PE P-20:0/20:5
Phosphatidylglycerols (10)	PG 16:0_16:0,PG 16:0_18:1,PG 16:0_20:3,PG 16:2_18:1,PG 17:0_18:1,PG 17:1_18:1,PG 18:1_18:1,PG 18:1_20:0,PG 18:1_20:1,PG 18:2_20:0
Phosphatidylinositols (4)	PI 16:0_16:0,PI 18:1_20:1,PI 18:1_20:2,PI 18:1_22:0
Phosphatidylserine	PS 36:1

**Table 3:** Lipids containing EPA or DHA changing during (9 weeks; serum) or after (serum, muscle, liver or brain) modGinA treatment

	ModGinA
**Serum (9 weeks)**	CE 20:5; CE 22:6; LPE 22:6; PE P-16:0/20:5; PE P-16:0/22:6; PE P-18:0/20:5; PE P-18:0/22:6; PE P-18:1/20:5; PE P-18:1/22:6; PE P-20:0/20:5; PG 16:0_20:5; PG 18:1_20:5; PG 18:2_20:5; PS 38:6
**Muscle**	*TG 22:6_34:1*; *TG 22:6_32:0*; *TG 22:6_34:2*; *TG* 22:6_32:1
**Liver**	PG 22:4_22:6, PE 42:7

Italics indicates also changing in GinA treatment (Table S2).

Next, we analyzed changes in metabolites that occurred between baseline and after 9 week treatment within each group (Table S4). We found that 268 metabolites changed in the vehicle, 186 in the GinA, and 316 in the modGinA groups. Of these, 121 changed in all three groups (Table S4). The change in the large number of metabolites in the vehicle group is not unexpected and is likely due to the daily administration of high oleic acid sunflower oil, which is known to have metabolic effects. Consistent with oleic acid administration, a large proportion of the lipid metabolites (157/241) were decreasing in vehicle, similar to what was observed in HL-7702 hepatocytes exposed to oleic acid (70 µM)^20^. For example, CE 22:6, Cer d18:1/22:0 and Hex-Cer d18:1/23:0 levels decreased in vehicle and after oleic administration in hepatocytes^20^. These changes appeared to be in part mitigated with either GinA or modGinA treatments (Table S4). Indeed, the majority of lipid metabolites increased following treatment with GinA (92/169) and modGinA (177/279).

Treatment with modGinA resulted in a significant increase in lactate (50%), anserine, GABA, alpha-AAA, and choline (see Table S4). In contrast, GinA treatment also led to an increase in choline, but to a lesser extent than with modGinA, alongside a decrease in β-alanine and threonine (refer to Table S4). Interestingly, threonine also decreased with vehicle but remained stable during modGinA treatment. Of interest, the majority of measured CEs changed with treatment, consistent with the observed decrease in circulating unesterified cholesterol levels with omega-3 polyunsaturated fatty acid treatment^21^. CE 18:0 and C18:1 increased in all groups. Ten CEs increased with modGinA, including CE 20:5 (Fig. 2D) and, of these, 3 decreased in vehicle while CE 14:1 decreased with both modGinA and vehicle (Table S4). A significant number of PLs changed during treatment in the 9th week, which is noteworthy because PLs play a crucial role in the synthesis of eicosanoids, potentially influencing the SASP phenotype^22^ (Table 2). Of the PLs, more PC and PC-O metabolites changed with vehicle (34) compared to either GinA (17) or modGinA (25), while the majority of PE-Ps changed with modGinA (23) compared to GinA (8) and vehicle (9). Similarly, PS metabolites predominantly increased in the modGinA (9) compared to the GinA (3) and vehicle groups (4). While a similar number of PGs, phosphatidyl inositols (PIs) and phosphatidic acids (PAs) changed in each group, as a whole PA metabolites showed a larger increase with GinA treatment. Changes in a large number of PAs are of interest, as PAs play an important role in membrane dynamics and cellular signaling, thereby regulating systemic inflammatory responses through the Akt/mammalian target of rapamycin/p70 S6 kinase 1 pathway^23,24^. The increase of choline and PA in circulation during treatment may result from increased phospholipase D (PLD) activation, an important effector enzyme that catalyzes the hydrolysis of PC to PA and choline (Lim et al.^23^). Interestingly, the modGinA group had a greater increase in choline while the GinA group had a greater increase in PAs.

Several lysolipids also changed during treatment, with lysophosphatidic acid (LPA) 18:2 decreasing in the modGinA group and lysophophatidyl glycerol (LPG) 16:1 down in all groups with the largest decrease in the GinA group. More lysophosphatidyl serines (LPS) and lysophosphatidyl cholines (LPC) changed in the modGinA group compared to the other groups, with the LPCs also mainly having the largest increase in the modGinA group followed by GinA (Table S4). Similar numbers of lysophosphatidyl ethanolamines (LPE) and lysophosphatidyl inositols (LPI) changed across groups.

Cers changed in all groups, with similar numbers of metabolites changing during treatment in all groups. The majority increased with GinA and modGinA and decreased in vehicle. Of interest, 11 of the 17 measured Hex-Cer changed in the modGinA group, while only 6 changed in the vehicle group and 4 in the GinA group (Table S4). Six SMs changed across all groups with larger changes in the GinA and modGinA groups, and SM 34:2 increased in the modGinA group only. Interestingly, several of the Cers and SMs contained nervonic acid (24:1), which increased in both treatment groups, with larger increases in the modGinA group (Table S4).

The differential changes observed in the concentrations of metabolites between GinA and modGinA treatment groups in the metabolomic data, may suggest that many such changes are concentration-dependent (C_max_), while others may require an increase in systemic exposure.

### Metabolomic analysis 10 days after treatment in skeletal muscle, liver, brain: Washout

In muscle, 115 metabolites were significantly different between groups after treatment by non-parametric one-way ANOVA, of which 109 metabolites were lipids (Table 4, Table S2). Aspartate, asparagine, betaine and glutamine increased with GinA treatment in muscle, and homocysteine decreased with GinA treatment (marginal decrease with modGinA) (Table S2). Citrulline increased in muscle with modGinA treatment (marginal increase with GinA). Citruline can improve mitochondrial function and secondarily improve and increase exercise capacity^25^. Of the lipids, eight acylcarnitines increased with GinA in muscle, of which only C12 was marginally elevated after modGinA treatment. Lysolipids LPE 14:1 and 22:5 were elevated in muscle following either treatment. Seventy-four PLs were predominantly increased in muscle after GinA, with eight increasing after modGinA treatment. Conversely, twelve Cers changed with treatment, of which 11/12 increased following modGinA treatment, and 3/12 increased following GinA treatment (Table 4; Table S2); Cer d18:1/23:0 and 26:0 had larger increases following modGinA treatment compared to GinA treatment group (Table S2). Fourteen glycerides (TGs) were associated with treatment, with 10 increased in both GinA and modGinA groups.

**Table 4:** A. Phospholipids that were significantly different 10 days after washout in mice receiving vehicle (high oleic sunflower seed oil), GinA (10 mg/kg) or modGinA (27 mg/kg) daily for 10 weeks in muscle, brain and liver (Kruskal- Wallis test). B. Ceramides that were significantly different in muscle, liver and brain by unadjusted Kruskal-Wallis test. C. Glycerides that were significantly different in muscle, liver and brain by unadjusted Kruskal-Wallis test (Table S3)

	Phospholipids
**A.**	
Muscle	PA 18:1_18:2; PA 18:2_20:1; PA 18:2_20:2; PA 18:2_22:3; PA 18:2_22:4; PC 32:0; PC 34:3; PC 38:4; PC 40:4; PC 40:5; PC 42:0; PC 42:5; PC 42:6; PC-O-34:1; PC-O-36:2; PC-O-36:3; PC-O-36:4; PC-O-38:2; PC-O-38:4; PC-O-40:4; PC-O-42:5; PE 30:1; PE 32:2; PE 34:4; PE 36:4; PE 38:2; PE 38:3; PE 38:4; PE 38:5; PE 40:4; PE 40:5; PE 40:6; PE 40:8; PE 42:7; PE 42:8; PE 44:6; PE 44:7; PE P-16:0/22:4; PE P-18:0/18:3; PE P-20:0/16:0; PG 16:0_16:0; PG 16:0_18:1; PG 16:0_18:2; PG 16:1_18:2; PG 16:1_20:4; PG 17:0_18:1; PG 18:0_18:1; PG 18:0_18:2; PG 18:0_18:3; PG 18:0_22:1; PG 18:1_18:2; PG 18:2_18:2; PG 18:2_20:5; PG 18:2_22:1; PG 18:2_22:4; PI 16:0_18:1; PI 16:0_18:2; PI 16:1_18:1; PI 17:1_18:2; PI 18:0_18:2; PI 18:0_20:0; PI 18:0_20:4; PI 18:0_22:0; PI 18:1_18:2; PI 18:1_20:3; PI 18:1_22:5; PI 18:2_20:0; PI 18:2_20:1; PI 18:2_20:4; PI 18:2_22:0; PI 18:2_22:6;PS 38:7; PS 40:5; PS 40:8
Brain	PE.P.18.1.20.5; PG.16.0_22.2
Liver	PG 22:4_22:6; PC O-42:0; PC O-38:1; PC 28:1; PA 18:2_20:0; PI 18:0_20:3; PI 17:0_18:1; PE 42:7
**B.**	
	Ceramides
Muscle	Cer d18:1/18:1; Cer d18:1/20:0; Cer d18:1/22:0; Cer d18:1/23:0; Cer d18:1/24:0; Cer d18:1/26:0; Hex2Cer d18:1/20:0; Hex2Cer d18:1/22:0; Hex-Cer d18:1/22:0; Hex-Cer d18:1/23:0; Hex-Cer d18:1/24:0; Hex-Cer d18:1/26:0
Brain	Cer.d18.2.24.0
Liver	Hex-Cer d18:2/24:0; Hex-Cer d18:1/22:0
**C.**	
	Glycerides
Muscle	TG 14:0_38:5; TG 16:0_40:6; TG 16:0_40:7; TG 17:2_36:2; TG 17:2_38:6; TG 20:4_32:0; TG 22:4_34:2; TG 22:5_32:0; TG 22:5_34:1; TG 22:5_34:2; TG 22:6_32:0; TG 22:6_32:1; TG 22:6_34:1; TG 22:6_34:2
Brain	MG.22.1; TG.22.4_34.2
Liver	DG 16:1_18:2

In brain, non-parametric one-way ANOVA identified 7 metabolites that were different between groups after treatment (Table 4; Table S2), of which 6 were lipids. Alpha-AAA decreased in the brain following GinA treatment with a marginal decrease with modGinA. Interestingly, alpha-AAA was elevated in the serum after 9 weeks of modGinA treatment. Cer d18:2/24:0 decreased following GinA treatment and both PLs trended down following GinA treatment (Table 4; Table S2). The acylcarnitine C3:1 decreased with modGinA treatment. Of interest, C3:1 was not different in muscle, but increased during GinA treatment in serum. Of the glycerides, MG 22:1 increased with modGinA, and TG 22:4_34:2 decreased with GinA. In muscle, TG 22:4_34:2 increased following GinA treatment (Table 4; Table S2).

In liver, 14 metabolites were different between groups after treatment by non-parametric one-way ANOVA (Table 4; Table S2), of which 11 were lipids. Alanine decreased with GinA, while betaine increased following modGinA treatment. Eight PLs changed in the liver with the majority increased with treatment (3/4 modGinA; 4/4 GinA). Hex-Cer d18:2/24:0 decreased following modGinA treatment and marginally decreased following GinA treatment. Of the glycerides, only DG16:1_18:2 increased after GinA treatment in the liver. In addition to the metabolomics assay, we also studied NAD^+^ metabolites in liver, as the liver is the central hub of metabolism and is a primary contributor to NAD^+^ biosynthesis^26^. Of the measured NAD^+^ metabolites, only NADP^+^ increased after modGinA treatment (Fig. S1). The significant increase of NADP^+^ over NAD^+^ may suggest increased NAD kinase activity, which may be associated with decreased oxidative stress^27^ and may suggest activation of the pentose phosphate pathway in the liver^28^.

### Senolytic effects

**Fig. 3:**
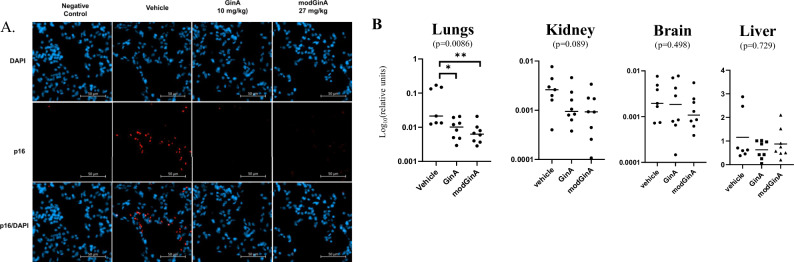
In vivo mouse model of doxorubicin-induced senescence. **A** Representative p16 immunofluorescent micrographs from lung of mice receiving vehicle (high oleic acid sunflower oil), 10 mg/kg GinA or 27 mg/kg of modGinA daily for 10 days prior to and 20 days after 10 mg/kg doxorubicin i.p. administration. The negative control group did not receive doxorubicin i.p. administration. Immunofluorescence analysis of colocalized signals for TdTomato (red; p-16) and nuclei stained with DAPI (blue). **B** Scatterplot of relative tdTomato staining over DAPI between groups (vehicle, GinA, modGinA) and different tissues (one-way ANOVA). Log base 10 scale on relative units. Significant differences between groups are indicated by *0.01 <*p* < 0.05; **0.001 <*p* < 0.01 (Mann–Whitney *U*-test).

In order to assess the senolytic effects of GinA and modGinA, we made use of an in vivo mouse model of doxorubicin-induced senescence in p16TdTom-expressing mice, in which expression of the fluorophore tdTomato is driven by the p16 promoter^29,30^. Mice were treated with vehicle, GinA or modGinA for 10 days prior to receiving doxorubicin and for 3 weeks thereafter. A representative micrograph visualizing p16 immunofluorescence in lung tissue (Fig. 3), revealed that tdTomato-expression is reduced with treatment (non-parametric one-way ANOVA *p* = 0.0086). A significant decline in the p16 levels (tdTomato expression) was observed for both the GinA (7.3% to 1.1%; *p* = 0.014) and modGinA (7.3% to 0.8%; *p* = 0.0022) treatment groups (Fig. S2; Table S5). In the kidney, an incremental decline of p16 expression was present between vehicle (0.3%) and treatment groups (GinA 0.15%; modGinA 0.12%), although these declines were not significantly different when analyzed by non-parametric one-way ANOVA (*p* = 0.089) or Mann–Whitney tests between groups. No substantial changes were detected in the brain or liver (Table S5).

### Behavioral Studies

As reductions in activity and motor function are observed with aging and have been associated with cellular senescence in animal models^31^, we tested the hypothesis that the application of a senolytic/senomorphic drug would improve motor function in aged mice, using a battery behavioral tests (see Methods) that are sensitive to aging. Locomotor activity in an open field chamber was unaffected by treatment throughout the full 30 min of testing (F2,33 = 0.08, *p* = 0.9) (Fig. 2E). Although conservative statistical analysis did not reveal significant effects of treatment in any of the behavioral tests conducted during the 10 days after washout (Fig. 2F), direct *t*-test comparison of vehicle and modGinA in trial 3 indicated a marginally significant effect (*p* = 0.0467) (Figure. S3). This may suggest the possibility that modGinA protects against age-related motor coordination, strength and/or balance decline. The lack of adverse events with either GinA or modGinA compared to vehicle suggests excellent safety profiles for both.

## Discussion

GinA has been shown to prevent local inflammation by inhibiting macrophage recruitment and regulating inflammatory cytokine expression in a mouse model (Suk et al., ^15^). We previously reported that GinA displayed senomorphic activity in vitro, where it reduced the secretion of SASP proinflammatory cytokines and increased the levels of cleaved caspase-3, a pro-apoptotic protein, in senescent fibroblasts^14^. In this study, during treatment we observed changes in circulating cytokine and metabolite levels indicating both pro- and anti-inflammatory effects. However, by the end of the study, there was a global reduction of the inflammatory milieu, with lower levels of IL-2, -5 and -6 in the modGinA treatment group. IL-5 is considered an important therapeutic target in asthma with several anti-IL-5 therapeutics (mepolizumab, reslizumab and benralizumab) commercially available (Farne et al.^32^). Since asthma is characterized by increased cellular senescence in bronchial fibroblasts and myofibroblasts^33^, the combined observations that modGinA resulted in decreased circulating IL-5 levels, and reduced pulmonary p16 expression in the senescence-induced mouse model suggest that modGinA could have potential therapeutic applications in chronic asthma. These findings should be verified in a mouse model of asthma, together with functional tests of the degree of bronchial obstruction with and without modGinA.

Treatment with modGinA and/or GinA resulted in changes that may result from changes in the mitochondria. For example, the increase of citrulline with treatment (GinA/modGinA) in muscle, can increase mitochondrial biogenesis and capillary density through the upregulation of peroxisome proliferator‐activated receptor‐gamma coactivator‐1α (PGC‐1α) in muscle^25^. Further, several mitochondrial derived PLs, including PE and PG, predominantly increased with treatment. PEs comprise ~30% of total mitochondrial PLs, are involved in regulating mitochondrial outer membrane permeability^34^, and play important roles in controlling membrane fusion, autophagy, and mitochondrial function^35^. PG and CDP-diacylglycerol are precursors for the de novo synthesis of the mitochondrial specific phospholipid nascent cardiolipin to tetra-linoleoyl cardiolipin, which is required for electron transport chain function and reduction of ROS^34^. The changes in PG, PE, PA and LPC suggest an increase in cardiolipin synthesis, comprised of two subunits of PA bridged through a glycerol backbone with four acyl chains^36^. While elevated levels of lactate with modGinA treatment suggests increased inflammation (Zhang and Lang, 2023), it can also point to increased skeletal muscle activity due to increased glycogen utilization, resulting in increased aerobic capacity and β-oxidation^37^.

In keeping with the changes in lactate, treatment with modGinA and/or GinA resulted in several changes suggesting enhanced TCA cycle activity and oxidative phosphorylation. The stability of threonine during modGinA treatment, for example, may indicate increased TCA cycle activity relative to GinA and vehicle *via* aspartate and oxaloacetate or fumarate as a precursor (Hao et al.^38^). Other changes include the citrulline-NO cycle interaction with the TCA cycle^39^; the increase of aspartate which can increase the TCA cycle intermediate malate via uptake of SLC1A3 transporters (Jin et al.^40^); the increase in glutamine observed with GinA; the increase of the TCA cycle intermediate succinate at 9 weeks with modGinA; and the increase of NADP^+^ in liver, which increases the catalysis of isocitrate to α-ketoglutarate via NADP^+-^dependent isocitrate dehydrogenases (IDHs). The α-Ketoglutarate, which increases in circulation as lactate increases, also has senomorphic activity as it reduced the SASP production in senescent fibroblasts and extended health and life spans in aged mice^41^. Interestingly, the increase in circulating acylcarnitines (Table 4A; Table S2) in muscle after GinA treatment may suggest increased cellular senescence^22^; however, a more likely hypothesis is that the increase is due to the inability of the TCA cycle activity to compensate for the increased β-oxidation rates due to increased fatty acid availability, as well as lactate and ketone bodies^42^. The increase in biomarkers of oxidative phosphorylation observed in both treatment groups is likely beneficial in senescence as defects in oxidative phosphorylation function have been observed in various models of senescence^43^, and may protect the mitochondria from functional deterioration^44^. The changes in the TCA cycle along with the changes observed in one-carbon metabolism across matrices (choline, betaine and homocysteine), suggest a role in purine synthesis. Several metabolites contribute to nucleotide de novo synthesis, including glucose, aspartate, glycine, and glutamine^45^, of which glucose, aspartate and glutamine changed with treatment in either serum and/or tissue. The loss of p16 expression promotes nucleotide synthesis at least in part *via* increased translation of the pentose phosphate pathway (PPP) enzyme ribose-5-phosphate isomerase A^45^ and is consistent with the reduction in p16 levels we saw in lung with modGinA.

Several changes in the metabolome observed with modGinA treatment favorably impact biochemical alterations that indicate positive effects on brain function, including cognitive function. Anserine (methylated derivative of carnosine) improves cerebral blood flow and cognitive function in animals and elderly human subjects^46,47^, choline counteracts brain aging and reduces cognitive decline with aging (Liu et al.^48^), and β-alanine can improve functional performance, delay fatigue and improve cognitive performance in older adults and reduce symptoms of depression^49^. Decreased alpha-AAA in the brain may be beneficial, as microinjection of alpha-AAA into the brain induced depressive-like behavior in mice (David et al.^50^). A reduction of CEs in the brain has been suggested to protect against Alzheimer’s disease (AD) like pathology^51^, and in our study, while not significant, after termination of treatment four CEs that we measured, CE14:0, 20:5, 22:2, 22:5 and 22:6, were decreased by the treatment. Furthermore, increasing PE levels were reported to reverse amyloid-β related cognitive deficits in a rat model of AD^52^. Nervonic acid (24:1) (NA) is essential for growth and maintenance of brain integrity and participates in the repair of damaged neuronal pathways^53^, and fish oil (rich in EPA, DHA and NA) improved the ability of mature oligodendrocytes to synthesize myelin proteins as well as SM^53^. Thus, our finding that modGinA treatment increased both NA containing Cer, SMs and Hex-Cer species, as well as EPA and DHA containing lipids in serum and muscle suggests potential benefits.

Previous studies suggested that GinA plays a role in regulating fatty acid metabolism and mitochondrial biogenesis by activating 5’ AMP-activated protein kinase (AMPK)^15^, a master regulator of metabolism. AMPK activation has been shown to be beneficial for cellular homeostasis and senescence prevention^54^ to promote fatty acid oxidation^55^. AMPK controls cell growth and several other cellular processes, including lipid and glucose metabolism, mitochondrial function, mitophagy and autophagy^55^. In our study, several of these metabolomic changes were observed, suggesting increased TCA activity and oxidative phosphorylation, as mentioned above, and AMPK activation. AMPK activation has also been shown to restore the NAD^+^ levels in senescent cells, thereby preventing oxidative stress-induced senescence because of improved autophagic flux and NAD^+^ homeostasis^54^. Notably, in our study NAD^+^ increased in the liver although the increase was not statistically significant. However, we detected an increase in NADP^+^ which is exclusively synthesized from NAD^+^ *via* cytoplasmic and mitochondrial NAD^+^ kinases. Omega-3 fatty acids (DHA, EPA) have been shown to elicit their anti-inflammatory effects *via* AMPK/SIRT1 pathways^56^ and, moreover, AMPK was found to reduce abnormal inflammatory responses and lung cellular senescence^57^. This is consistent with the significant decrease in the number of senescent cells seen in lung with modGinA treatment.

Several limitations exist with the study, including the exploratory nature of the study due to the small sample size, and as a result no adjustments for multiplicity were made, the requirement of high oleic sunflower oil as vehicle and the daily administration of drug by oral gavage. The overall strengths include the study in two models: aged mice (79–80 weeks old), and an in vivo mouse model of doxorubicin-induced senescence; the metabolomics and histology carried out across multiple compartments within the same mouse.

## Methods

### Materials

Gingerenone A was purchased from Aobious (Gloucester, MA). Cytokines were measured by multiplex assays V-Plex plus Proinflammatory panel 1 mouse kit from Mesoscale Diagnostics (Rockville, MD).

### Synthesis of modified Gingerenone A (modGinA)

(An equimolar mixture of GinA-DHA and GinA-EPA) (Fig. 1).

#### Gingerenone A DHA (GinA-DHA)

A solution of DHA (458 mg; 2.4 equiv.) in 3 ml of dichloromethane was added to a solution of Gin A (207 mg) in 8 ml of dichloromethane, followed by 4-dimethylaminopyridine (14 mg; 0.2 equiv.). The mixture was cooled to 0 °C, then dicyclohexylcarbodiimide (383 mg; 3.2 equiv.) was added. The reaction was stirred at 0 °C for 10 min, then at room temperature overnight.

The mixture was first concentrated and then 10% ethyl acetate/hexane (E/H) was added and the mixture was filtered through a pad of 1:1 silica:Celite, eluting with 15% E/H. The solvent was evaporated and the crude mixture was purified on a 40 g silica column (RediSep R_f_ flash cartridge) using a gradient of 10%-15%-25% E/H to elute the product. All fractions containing the di-ester product (Rf = 0.45) were combined and evaporated to yield 305 mg of pure GinA-di-DHA ester (54% yield). 1H NMR (400 MHz, Chloroform-d) δ 6.98 – 6.75 (m, 7H), 6.16 (dt, J = 15.9, 1.5 Hz, 1H), 5.56–5.28 (m, 24H), 3.82 (s, 6H), 2.98–2.82 (m, 22H), 2.79 (dd, J = 15.9, 8.6 Hz, 3H), 2.66 (t, J = 7.4 Hz, 4H), 2.61 – 2.49 (m, 7H), 2.16–2.04 (m, 4H), 0.99 (t, J = 7.5 Hz, 6H). NMR data was obtained on a 400 MHz Bruker Avance NMR spectrometer equipped with a 5 mm BBO probe. A standard Bruker parameters set was employed with 64k data points, 16 scans, and a relaxation delay of 1 s. TLC was visualized with UV and with sulfuric acid spray plus heating.

#### Gingerenone A EPA (GinA-EPA)

A solution of EPA (837 mg; 2.4 equiv.) in 5 ml of dichloromethane was added to a solution of Gin A (411 mg) in 20 ml of dichloromethane, followed by the addition of 4-dimethylaminopyridine (28 mg; 0.2 equiv.). The mixture was cooled to 0 °C, then dicyclohexylcarbodiimide (761 mg; 3.2 equiv.) was added. The reaction was stirred at 0 °C for 10 min, then at room temperature overnight.

The mixture was concentrated then 10% ethyl acetate/hexane (E/H) was added and it was filtered through a pad of 1:1 silica:Celite, eluting with 15% E/H. The solvent was evaporated and the crude mixture was purified on a 80 g silica column (RediSep Rf flash cartridge) using a gradient of 10%–15%–25% E/H to elute the product. All fractions containing the di-ester product (Rf = 0.45) were combined and evaporated to yield 800 mg of pure GinA-di-EPA ester (75% yield). 1H NMR (400 MHz, Chloroform-d) δ 7.00–6.70 (m, 7H), 6.16 (dt, J = 15.9, 1.5 Hz, 1H), 5.52–5.28 (m, 19H), 3.88–3.75 (m, 6H), 2.98–2.81 (m, 19H), 2.78 (dd, J = 9.0, 6.6 Hz, 2H), 2.65–2.49 (m, 6H), 2.24 (q, J = 6.8 Hz, 4H), 2.17–2.04 (m, 4H), 1.86 (p, J = 7.4 Hz, 4H), 1.00 (t, J = 7.5 Hz, 6H). NMR data was obtained on a 400 MHz Bruker Avance NMR spectrometer equipped with a 5 mm BBO probe. A standard Bruker parameters set was employed with 64 k data points, 16 scans, and a relaxation delay of 1 s. TLC was visualized with UV and with sulfuric acid spray plus heating.

### Mice

All mouse work, including the import, housing, experimental procedures, and euthanasia, followed a detailed animal study protocol approved by the Animal Care and Use Committee (ACUC) of the National Institute on Aging (NIA) Intramural Research Program (IRP), fully accredited by the American Association for Accreditation of Laboratory Animal Care (AAALAC). C57BL/6JN mice came from the NIA Aged Rodent Colonies and were acclimatized to housing at the NIA animal facility for at least 1 week prior to experimentation. For the 10 week study, the C57BL/6JN mice were between 79 and 80 weeks old. p16tdTom mice were originally developed by the Sharpless lab^30^.

All mice were maintained on a standard diet with food and water available ad libitum. Animals were maintained on a 12 h light/dark cycle, with all testing performed during the light cycle. The animals were group housed whenever possible.

### Systemic exposure

40 C57BL/6JN Mice (19 months old), averaging 26 g(+/-2g) from the NIA Aged Colony received molar equivalents of GinA (10 mg/kg) or modified GinA (1:1 GinA-DHA: GinA-EPA; (27 mg/kg)) administered by oral gavage. Serum was collected post-administration at multiple timepoints (0.25 h, 0.5 h, 1 h, 2 h and 4 h) and circulating levels of GinA were determined in both groups.

### Chronic treatment of GinA or modGinA

Forty-three aged C57BL/6JN mice (79–80 weeks old) were administered either high oleic sunflower oil (vehicle) (14 mice), 10 mg/kg/day GinA (from 1.8 mg/ml stock solution) (14 mice) or 27 mg/kg/day modGinA (from 4.8 mg/ml stock solution) (14 mice) by oral gavage (p.o.) once daily for 10 weeks. Serum was collected by mandibular bleed after 9 weeks of treatment as opposed to 10 weeks to avoid the expected weight loss during washout. At the end of the study (10 days after administration of the last dose) the remaining animals were euthanized (at 98/99 weeks) via excessive carbon dioxide inhalation. Following cessation of breath, a terminal cardiac blood draw was done. The blood was transferred to serum collection tubes, placed at room temperature for 30 mins and then centrifuged at 4 °C for 10 mins at 2000 x g. The serum samples were then frozen at −80 °C. Immediately following blood collection, the whole brain was dissected and placed on dry ice. Muscle samples were collected from the gastrocnemius and liver was collected and placed on dry ice. Samples were later transferred to a -80 °C freezer.

### Behavioral studies

Mice were familiarized to human handling at least 3 days prior to testing. Behavioral studies were conducted exclusively during the washout period to avoid interference from ongoing oral gavage administration.

#### Rotarod

Rotarod was carried out as previously described^58^. Briefly, mice were placed on a 3.2 cm diameter drum (MED-Associates; St Albans, VT) rotating at 4 RPM and allowed 10 s of acclimation. During this time any mice that fell were replaced atop the drum. Rotation then accelerated to 40 RPM over a 5 min testing period, and latency to fall was detected via infrared beam. Mice were tested for three trials with a minimum intertrial interval of 60 min.

#### Open field

Mice were placed in a 44 × 44 x 30 cm clear Plexiglas arena (MED-Associates; St Albans, VT) enclosed in a dimly lit sound attenuating cubicle. Movements were detected by arrays of infrared beams for 30 min with use of Activity Monitor software (MED-Associates; St Albans, VT).

#### Grip strength

Mice were allowed to grab with their forelimbs onto a metal T-bar affixed to a grip strength meter (Bioseb; Pinellas Park, FL). Once secure, the mouse was pulled by the tail until it let go of the bar. The maximum value obtained from 5 trials was recorded.

#### Wire hang

Mice were placed atop a wire cage-top that was inverted to allow mice to hang upside down while grasping with all four limbs. If they fell off within 10 s, they were placed back on the cage-top and time was reset. If the mouse fell off three times prior to 10 s, the longest of the three was recorded. Latency to fall was recorded up to a maximum of 10 min for three trials, with intertrial intervals of at least 30 min. Log10 transformation on fall latencies was used to produce a normal distribution.

#### Home-cage monitoring

Mice were individually housed in a Tecniplast Digital Ventilated Caging system that continuously recorded activity using a grid of capacitive sensors beneath each cage^59^. Light cycling and ad libitum access to food and water were as normal. After 3 days of activity recording, animals were returned to group cages.

### GinA quantification

Ten µl of the internal standard naringenin (200 ng/ml) in acetonitrile and 2 µl of acetonitrile were added to 60 µl of serum. The solution was mixed on a Thermomixer for 1 min at 1000 rpm at room temperature. Then 150 µl of acetone followed by 150 µl of acetonitrile and 450 µl of ethyl acetate were added and the solution was Thermomixed at 1000 rpm for 10 min at room temperature. The solution was centrifuged at 17,000 x g for 10 min at 4 ^o^C. The supernatant was removed and stream dried under nitrogen. The precipitate was reconstituted with a solution of 30 µl of acetonitrile:water (1:1) containing 0.1% formic acid. The solution was Thermomixed for 2 min at 1000 rpm at room temperature, and then added to an autosampler vial for analysis and 15 µl was injected. GinA analysis was accomplished using a Phenomenex Security Guard C18 (4 × 3 mm ID) and an XBridge C18 3.5 µm (2.1 mm × 100 mm). The mobile phase consisted of 5 mM ammonium formate and 0.1% formic acid as component A and acetonitrile as component B. A linear gradient was run as follows: 0 min 10% B; 1 min 10%B, 5 min 90% B; 10 min 90% B; 10.1 min 10% B at a flow rate of 0.2 mL/min, with an oven temperature at 40 °C. The total run time was 15 min per sample. The MS/MS analysis was performed using a QTRAP mass spectrometer model API 5500 system from Applied Biosystems/MDS SciEx in negative ionization mode equipped with Turbo Ion Spray® (TIS) (Applied Biosystems, Foster City, CA, USA). The MRMs were: MRM1 (355.11-219.10) and MRM2 (355.11-82.90) for GinA; and MRM1 (271.10-150.91) and MRM2 (271.00-106.900) for the internal standard naringenin. The TIS instrumental source settings for temperature, curtain gas, ion source gas 1 (nebulizer), ion source gas 2 (turbo ion spray) and ion spray voltage and entrance potential were 400 °C, 25 psi, 60 psi, 60 psi and -4500 V and -10V, respectively. For GinA TIS compound parameter settings (declustering potential, collision energy and cell exit potential) for MRM1 were -105 V, -20 V and -11 and for MRM2 were -105 V, -32 V and -7. For naringenin MRM1 was -100 V, -24 V and -10 V, and MRM2 was -100V, -33 V and -10 V. The quantitation of GinA was accomplished with a seven-point calibration curve (12.5 to 0.097 ng/ml). The data were acquired and analyzed using Analyst version 1.4.2 (Applied Biosystems).

### Targeted metabolomics

A targeted metabolomic assessment was carried out following the manufacturer’s protocol using Biocrates’ MxP® Quant 500 XL kit (www.biocrates.com; Biocrates, Innsbruck, Austria), in mouse serum (10 µl), mouse muscle (10 µl), mouse brain (10 µl) and mouse liver (10 µl) following previously described protocol^60^. For the muscle, brain and liver, tissue was homogenized in 2 ml tubes from the Precellys Lysing Kit, soft tissue homogenizing CK14 (Bertin, Rockville, MD) using 3 µl/mg of Isopropanol. The tubes were then homogenized for 3 cycles of 20 s at 5500 rpm with 30 s interval between the homogenization steps using the Precellys 24 between 0 and 4 °C. For liver tissue, 6000 rpm by 30 s x 2 on Precellys Evolution. Afterwards the tubes were centrifuged with a benchtop centrifuge and the solution was transferred to a separate 1.5 ml Eppendorf tube. The tubes were then centrifuged for 5 mins at 10,000-x g in the cold room and the samples were stored at −80 °C until analysis.

The Biocrates MxP 500XL was run using a Nexera HPLC system (Shimadzu) coupled to a 6500 QTRAP® mass spectrometer (AB Sciex) with an electrospray ionization source as previously described (13). Data were quantified using WebIDQ™ software and normalized to internal quality controls. Any metabolite that had greater than 30% above limit of detection (LOD) was excluded, while those with less than 30% LOD had their values imputed as 1/5 the lowest concentration. If the LODs if were predominantly restricted to one group and therefore would be >30% above LOD, those would still be considered. After exclusions, 736 metabolites were detected in serum at baseline, 745 metabolites at 9 weeks, and 756 metabolites 10 days after termination of treatment. In muscle, 698 metabolites were detected, 544 metabolites in brain and 769 metabolites in liver.

### NAD^+^ metabolome in liver

Liver samples were prepared and diluted at a concentration of 20 mg tissue/500 µl 80:20 (methanol:water) solution. The diluted liver samples were homogenized using Precellys Evolution at 6500 rpm, 2 × 20 s cycle with pause of 30 s. Homogenized samples were heated and mixed in a thermomixer at 80 °C for 3 min at 1000 rpm and then centrifuged at 17000 x g for 20 min at 4 °C. 50 µl of supernatant from centrifuged samples was pipetted to separate Eppendorf tube along with 5 µl of internal standard (Dry extract of >2 × 10^9^ Pichia pastoris cells (~15 mg), (Metabolite Yeast Extract (U-13 °C, 98%)) dissolved in 2 mL of deoxygenated buffered ethanol (75% ethanol/25% 1 mM HEPES, pH 7.1)). This mixture was vortexed for 1 min and centrifuged on benchtop centrifuge for an additional minute. The supernatant was then transferred to an autosampler vial for analysis on the LC MS/MS using a previously developed method^61^. Precision is reported (Table S6).

### Protein analysis V-PLEX

The V-PLEX Plus Proinflammatory Panel 1 Mouse Kit was used following the manufacturer’s protocol (Mesoscale Diagnostics) to carry out cytokines/chemokines analyses in mouse serum collected during treatment (at 9 weeks) and serum, muscle and brain collected 10 days after the last dose. Baseline samples were also collected, however, while there were sufficient blood volumes for the targeted metabolomics, there was only sufficient volumes in 16 samples (4 vehicle, 6 modGinA and 6 GinA treatment group) for the V-Plex kit. For the muscle and brain tissue samples, 10ul of RIPA buffer (Thermoscientific #89901) was added per mg of tissue. Halt protease/ phosphatase inhibitor cocktail and Leupeptin A were added to stock RIPA buffer. Muscle and brain tissue were homogenized in 2 ml Precellys tubes (Bertin) at 6000 rpm for 2 rounds of 30 s. Tubes are incubated at 37 °C with shaking for 1 h at 500 rpm. Samples were then centrifuged at 12,000 x g for 20 min. Supernatant was used for MSD assay kit and BCA protein analysis. 25ul of supernatant was used for MSD assay kit and 50ul for duplicate BCA protein analysis. The remainder was frozen at -80 °C. Samples and controls were diluted 2-fold within each well; standards were serial diluted in separate tubes then pipetted onto the MSD plate. The plate was then sealed and incubated and put on a microplate shaker (700 rpm) for 2 h at room temperature. Plates were then washed 3x and the corresponding detection antibody solution was added to each well. The plate was sealed and again incubated for 2 h at room temperature with shaking at 700 rpm. The plates were washed three times and Read Buffer T was added to each well. The plates were immediately read on the MESO Quickplex SQ 120MM, Model 1300, (MSD, Rockville, MD) with the Methodical Minds software version MMPR 1.0.38. Protein concentrations were determined using MSD Discovery workbench 4.0.

### In vivo mouse model of doxorubicin-induced senescence

Twenty-eight heterozygous male p16TdTom knock-in mice, which express tdTomato from the endogenous p16Ink4 gene, aged between 8 and 12 weeks, were acclimated to housing at the National Institute on Aging (NIA) Intramural Research Program for at least one month prior to experimentation. Two weeks before the start of treatment administration, serum was collected via mandibular bleed.

The mice were assigned to one of three treatment groups: high oleic sunflower oil (vehicle; *n* = 7), 10 mg/kg GinA (*n* = 8), or 27 mg/kg modGinA (*n* = 8). Treatments were administered once daily via oral gavage (p.o.) for 10 days. Following this period, all treatment groups, received a single intraperitoneal injection of doxorubicin (10 mg/kg) dissolved in 10% DMSO in sterile PBS (50 µl), as described by Anerillas et al. ^29^. An additional five vehicle-treated mice received only the 10% DMSO in sterile PBS solution. The treatment regimen (vehicle, GinA, or modGinA) continued for approximately 20 days following doxorubicin administration.

One day after the final treatment, terminal cardiac blood was collected. For histological analysis, mice were perfused with PBS followed by 4% paraformaldehyde (PFA) in PBS. The brain, lungs, liver, and kidneys were harvested and post-fixed in 4% PFA overnight at 4 °C before being processed for paraffin embedding using a tissue processor (Leica HistoCore PEARL). Paraffin blocks were sectioned at a thickness of 10 µm (lung, kidney, brain) or 5 µm (liver).

Following deparaffinization, antigen retrieval was performed using citrate buffer (pH 4.0, Sigma). For lung sections, antigen retrieval involved incubation in a 90 °C water bath for 30 min followed by cooling at room temperature for 2 h. For kidney and brain sections, antigen retrieval was performed using an Aptum 2100 Retriever, followed by cooling at room temperature for 2 h. Sections were blocked with 5% donkey serum for 1 h and incubated overnight at 4 °C with a primary antibody against tdTomato (Cell Signaling). After washing, sections were incubated with an AlexaFluor 555-conjugated secondary antibody for 2 h at room temperature. Slides were mounted using ProLong Gold Antifade Mountant with DAPI (Life Technologies).

Images were captured using a Zeiss Axiovert 7 Observer fluorescence microscope equipped with a 20x (0.8) magnification lens. Red and blue fluorescence channels were analyzed using ImageJ software. The channels were converted into binary images, and the number of events was quantified using the particle analysis feature. The total red area was normalized to the total blue area for each sample to generate a quantitative measure of tdTomato expression.

### Data processing and statistical analysis

Processed, quality control (pooled plasma samples provided by Biocrates) normalized data were log transformed and metabolites with >30% missing data were excluded from analysis. Any metabolite with >30% missing samples were excluded from analysis and metabolites with <30% missing values but concentrations below the limit of detection and were imputed by 1/5 of minimum positive values for each variable. Proteins that had greater than +3 SD from the z-score mean were excluded from the analysis. For the protein and metabolomic analysis in serum and tissues, an unadjusted non-parametric ANOVA (Kruskal–Wallis test) analysis was used between the three groups (vehicle, GinA, modGinA), followed by a Dunnett’s test comparing treatment groups to vehicle. The longitudinal serum metabolomic analysis spanned three time points, namely T0 (baseline), T1 (at the 9th week of treatment) and T2 (10 days after washout). For each metabolite, a mixed-effects model was run according to the formula “conc ~(1|subj) + tp + g + tp*g”, where the metabolite’s concentration (conc) was regressed against fixed effects for time point (tp), group (g) and their interaction (tp*g), considering subject identifiers (subj) as random effects. The statistical significance of the interaction term was assessed by performing a likelihood ratio test to compare the full model against the null model: “conc ~(1|subj) + tp + g” using the ANOVA function from R package stats v.4.4.0. Mixed-effects models were implemented in a script via the lmer function from R package lme4 v.1.1.35.5 with the argument REML = FALSE to optimize the log-likelihood^62^. Supplementary Table S3 includes annotations to describe whether each model was singular or not (Y = Yes, N = No). A fitted mixed model is singular if the parameters are on the boundary of the feasible parameter space; see lme4 documentation for details. A complementary analysis based on Wilcoxon tests was performed to compare group pairs, as follows: (i) pairwise comparison of time points, performed separately for each treatment group; (ii) pairwise comparison of treatment groups, performed separately for each time point; and (iii) pairwise comparison of treatment group changes, performed separately for each pair of time points. The first comparison was done with a paired Wilcoxon test, because we compared two sets of measurements performed on the same mice at different time points; the remaining comparisons were unpaired.

## Supplementary information

Supplemental Figures_Tables

Supplemental Table 4

## Data Availability

No datasets were generated or analysed during the current study.
