# Publisher Correction: Senolytic effects of a modified Gingerenone A

**DOI:** 10.1038/s41514-025-00246-9

**Published:** 2025-06-23

**Authors:** Ruin Moaddel, Chad Sanehira, Gregory Keyes, Chang-Yi Cui, Reza Ahmadkhaniha, Julián Candia, Nathan L. Price, Sarah Eckroth, Bryce Middleton, Mohammed Khadeer, Caio H. Mazucanti, Ross A. McDevitt, Myriam Gorospe, Rafael de Cabo, Josephine M. Egan, Christopher E. Ramsden, Luigi Ferrucci

**Affiliations:** 1 Laboratory of Clinical Investigation, National Institute on Aging, Intramural Research Program, NIH, Baltimore, MD USA; 2 Translational Gerontology Branch, National Institute on Aging, Intramural Research Program, NIH, Baltimore, MD USA; 3 Laboratory of Genetics and Genomics, National Institute on Aging, Intramural Research Program, NIH, Baltimore, MD USA; 4 Comparative Medicine Section, National Institute on Aging, Intramural Research Program, NIH, Baltimore, MD USA

**Keywords:** Chemical biology, Drug discovery, Therapeutics

Correction to: *npj Aging* 10.1038/s41514-025-00230-3, published online 30 May 2025

In this article the author name ‘Ahmadkhania’ was incorrectly written and should have been ‘Ahmadkhaniha’. The original article has been corrected.

