# Supplementary material for: Senolytic effects of a modified Gingerenone A
Source: NPJ Aging. 2025 May 30;11(1):45. doi: 10.1038/s41514-025-00230-3 (PMC12125167; doi:10.1038/s41514-025-00230-3)
Supplement: Supplementary file 1 — Supplemental Figures_Tables [file 41514_2025_230_MOESM1_ESM.pdf]

Figure S1. Levels of NAD^+ and NAD^+ metabolites in liver tissue collected 10 days after termination of treatment of a daily oral administration of GinA (10 mg/kg) or modGinA (27 mg/kg) or vehicle (high oleic acid sunflower oil) in ~80-week old C57BL/6JN mice for 10 weeks. A. Separation of the NAD^+ metabolites in standard solution using an Accucore HILIC column (2.1 × 150 mm, 2.6 µm, Thermo) at 32 °C with a chromatographic run time of 14 min. The chromatogram is presented with intensity in cps (y-axis) and time (min) on the x-axis. B. Levels of NAMN, AcCoA and $NADP^+$ * ($p<0.05$) and · ($0.05<p<0.10$).

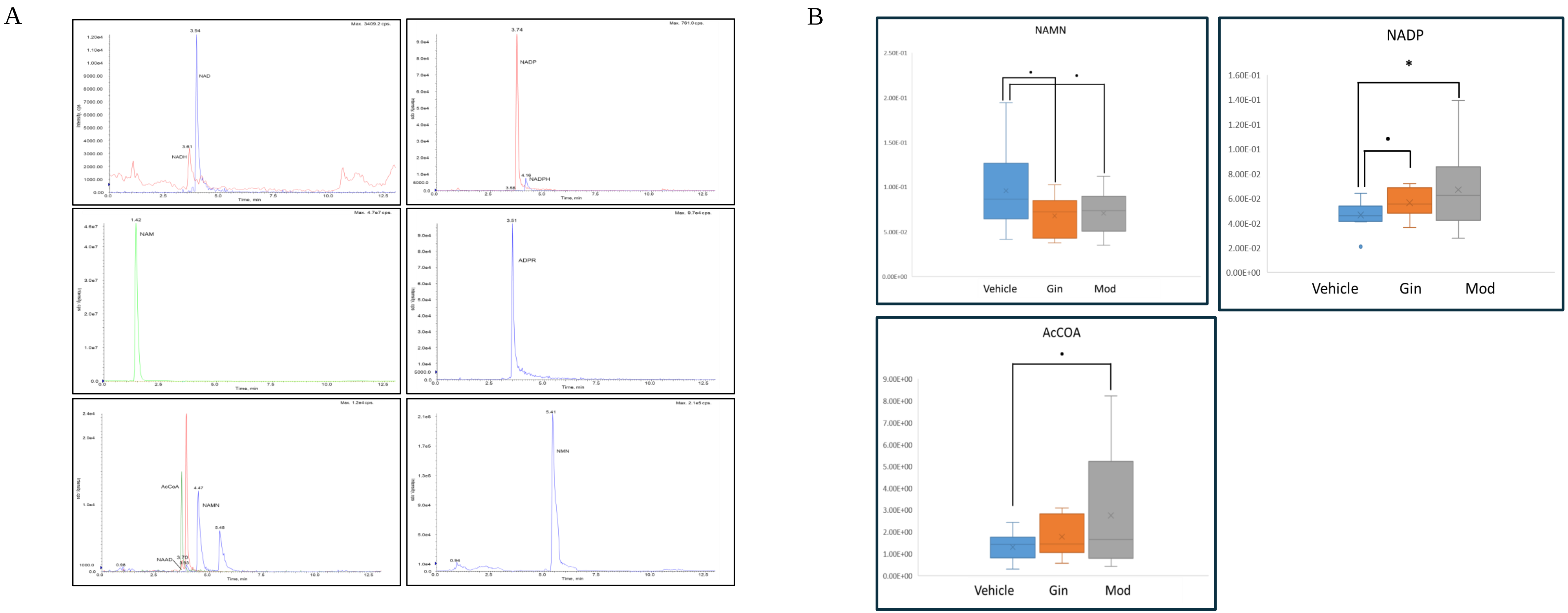

Figure S2. Representative p16 immunofluorescent full micrographs from lung in the indicated groups receiving vehicle (high oleic acid sunflower oil), 10 mg/kg GinA or 27 mg/kg of modGinA daily for 10 days prior to and 20 days after 10 mg/kg doxorubicin i.p. administration. Immunofluorescence analysis of colocalized signals for TdTomato (red; p-16) and nuclei stained with DAPI (blue) in an *in vivo* mouse model of doxorubicin-induced senescence.

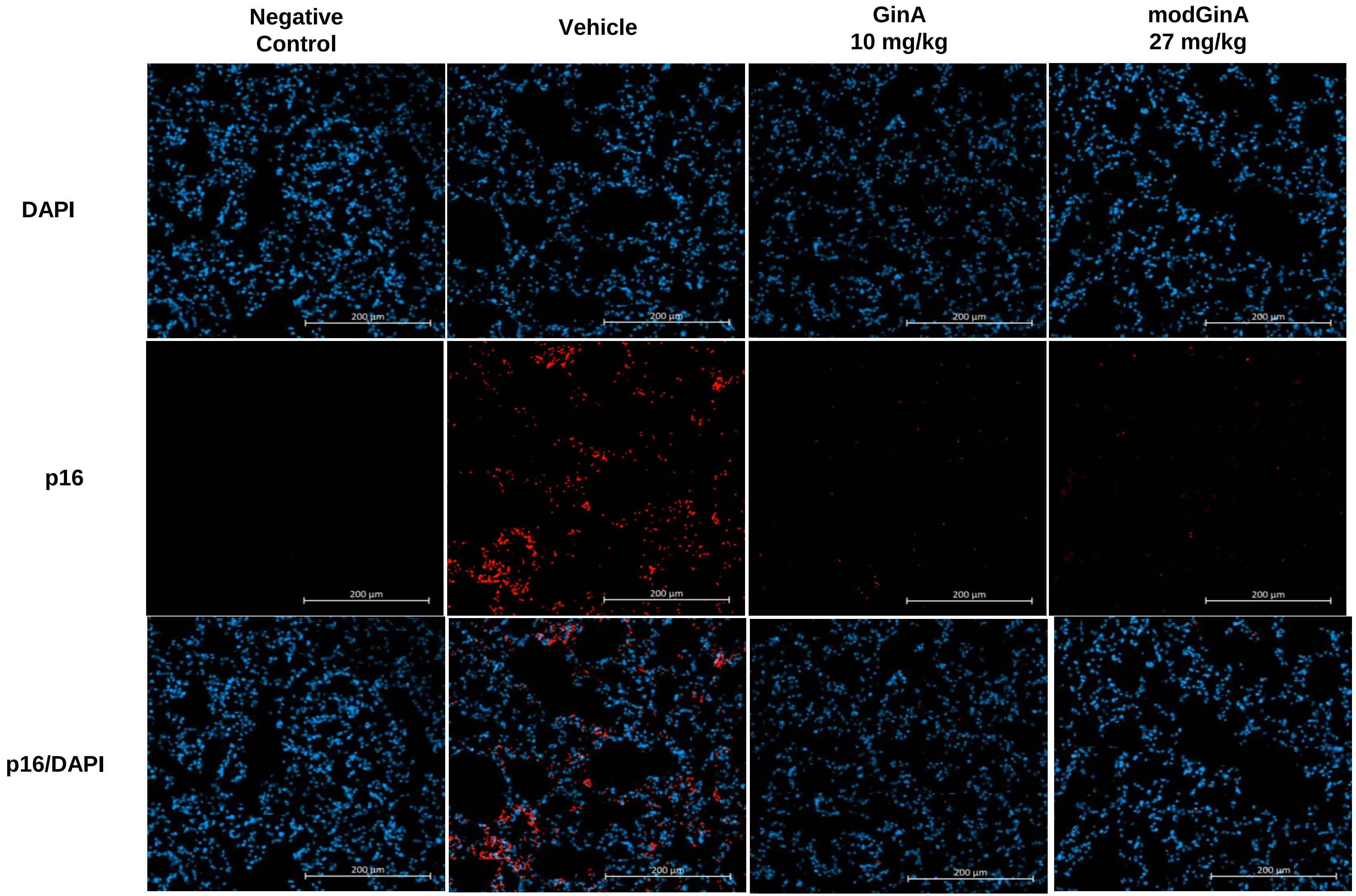

Figure S3. Rotarod experiments carried out on aged C57BL/6JN mice 8 days after receiving a daily administration of high oleic sunflower oil (vehicle (grey)), 10 mg/kg GinA (blue) and 27 mg/kg modGinA (red). Latency to fall is shown for each trial. In trial 3 of modGinA latency to fall was significantly higher compared to vehicle (unpaired t-test p=0.0467).

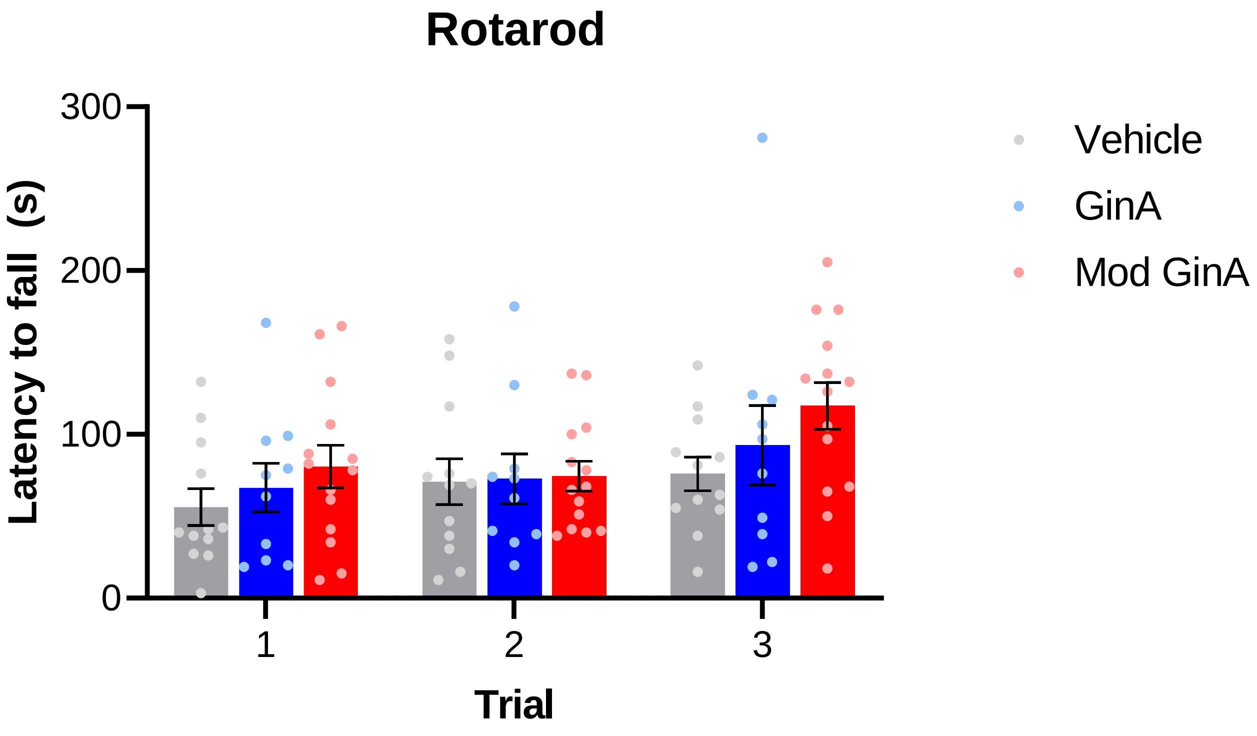

Table S1. **A.** Cytokine levels in pg/ml in serum at baseline prior to treatment in mice in the vehicle group (n=4), modifiied GinA group (n=6) and GinA group (n=6) . Proteins that were significantly different by non-parametric one-way anova analysis (Kruskal-Wallis test) are in red font (p-values are in paranthesis). Concentration is in blue were significant in follow-up Dunnetts test comparing treatment groups to vehicle. **B.** Cytokine levels in pg/ml in liver, brain and muscle in mice receiving high oleic sunflower oil (vehicle) (n=12), 27 mg/kg modGinA (n=14) or 10 mg/kg GinA (n=10) or daily for 10 weeks. Tissues were collected 10 days after treatment cessation (Post treatment). Proteins that were significantly different by non-parametric one-way anova analysis (Kruskal-Wallis test) are in red font (p-values are in paranthesis). Concentration is in blue were significant in follow-up Dunnetts test comparing treatment groups to vehicle.

A.

	**Serum (Baseline)**		
**Protein** (KW p-values)	**vehicle**	**GinA** (p-value)	**modGinA** (p-value)
IFNγ (0.4185)	0.67 ± 0.86	0.605 ± 0.414 (0.9733)	0.28 ± 0.078 (0.3879)
IL-10 (0.3808)	5.54 ± 0.76	8.44 ± 5.76 (0.4071)	5.60 ± 2.24 (0.9995)
IL-1β (0.6195)	0.41 ± 0.13	0.57 ± 0.59 (0.7541)	0.34 ± 0.19 (0.9493)
IL-2 (0.1572)	0.53 ± 0.40	0.70 ± 0.27 (0.5480)	0.36 ±0.21 (0.5545)
IL-5 (0.1355)	1.68 ± 0.34	1.43 ± 0.48 (0.9319)	2.98 ± 2.05 (0.2414)
IL-6 (0.5700)	11.21 ± 8.95	10.46 ± 5.06 (0.9914)	17.32 ± 16.62 (0.6233)
CXCL1 (0.1166)	34.76 ± 8.41	44.80 ± 10.45 (0.141)	46.52 ± 3.82 (0.0944)
TNFα (0.4387)	5.07 ± 0.95	6.26 ± 0.77 (0.3286)	5.77 ± 1.99 (0.6482)

B.

	**Liver**		
**Protein** (KW p-values)	**vehicle**	**GinA** (p-value)	**modGinA** (p-value)
IFNγ (0.1706)	0.29 ± 0.15	0.33 ± 0.18 (0.7995)	0.22 ± 0.08 (0.3214)
IL-10 (0.9358)	12.40 ± 2.08	12.35 ± 2.34 (0.9983)	12.67 ± 2.60 (0.9406)
IL-1β (0.1567)	30.63 ± 20.40	20.01 ± 7.62 (0.1338)	22.36 ± 8.46 (0.2218)
IL-2 (0.5708)	1.35 ± 0.35	1.47 ± 0.46 (0.6928)	1.30 ±0.38 (0.9272)
IL-5 (0.7574)	0.93 ± 0.21	1.01± 0.23 (0.6877)	0.98 ± 0.30 (0.8389)
IL-6 (0.3628)	41.32 ± 7.73	45.75 ± 12.62 (0.4739)	40.03 ± 9.09 (0.9222)
CXCL1 (0.0698)	6.66 ± 5.48	4.60 ± 2.50 (0.3091)	**3.26 ± 1.10** (0.0417)
TNFα (0.2708)	2.12 ± 1.18	1.86 ± 0.92 (0.7057)	1.56 ± 0.36 (0.1902)

	**Brain**		
**Protein** (KW p-values)	**vehicle**	**GinA** (p-value)	**modGinA** (p-value)
IFNγ (0.0692)	0.12 ± 0.07	0.06 ± 0.03 (0.0502)	0.11 ± 0.05 (0.8064)
IL-10 (0.4128)	0.61 ± 0.55	0.92 ± 0.58 (0.3197)	0.71 ± 0.49 (0.8467)
IL-1β (0.9362)	4.83 ± 1.48	5.08 ± 1.76 (0.9174)	4.87 ± 1.85 (0.9962)
IL-2 (0.3538)	0.50 ± 0.32	0.36 ± 0.24 (0.3111)	0.39 ± 0.16 (0.4120)
IL-5 (0.9931)	0.65 ± 0.35	0.65 ± 0.42 (0.9999)	0.67 ± 0.45 (0.9914)
IL-6 (0.5808)	7.45 ± 3.20	6.34 ± 2.24 (0.5516)	6.49 ± 2.72 (0.5892)
CXCL1 (0.2980)	5.70 ± 1.42	6.02 ± 1.89 (0.8856)	4.95 ± 1.80 (0.4778)
TNFα (0.1338)	0.37 ± 0.21	0.40 ± 0.19 (0.1657)	0.42 ± 0.34 (0.9845)

	**Muscle**		
**Protein** (KW p-values)	**vehicle**	**GinA** (p-value)	**modGinA** (p-value)
IFNγ (0.9395)	0.041 ± 0.049	0.048 ± 0.06 (0.9629)	0.038 ± 0.07 (0.98844)
IL-10 (0.3945)	0.23 ± 0.34	0.52 ± 0.45 (0.4371)	0.60 ± 0.82 (0.3641)
IL-1β (0.8787)	0.94 ± 0.81	1.16 ± 1.83 (0.8941)	1.67 ± 3.10 (0.9935)
IL-2 (0.7587)	0.15 ± 0.17	0.21 ± 0.22 (0.7337)	0.16 ±0.24 (0.9996)
IL-5 (0.4642)	0.33 ± 0.48	0.22 ± 0.28 (0.7078)	0.17 ± 0.23 (0.3652)
IL-6 (0.5411)	4.41 ± 3.19	4.22 ± 2.87 (0.9825)	3.24 ± 2.47 (0.4825)
CXCL1 (0.0766)	0.97 ± 0.28	1.00 ± 0.61 (0.9891)	0.66 ± 0.31 (0.1044)
TNFα (0.7949)	0.49 ± 0.59	0.65 ± 0.89 (0.794)	0.47 ± 0.53 (0.9984)

Table S2 Metabolites that were significantly different ($p<0.05$;unadjusted) by non-parametric one-way anova analysis (Kruskal-Wallis test) in mice receiving vehicle (high oleic sunflower seed oil) (v), GinA (10 mg/kg) (g) or modGinA (27 mg/kg) (m) daily for 10 weeks. Circulating concentrations in serum at baseline (A), at the 9th week during treatment (B), 10 days after washout (C) and in muscle (D), brain (E) and liver (F) 10 days after washout are reported.

A. Baseline

Column1	ANOVA_p.val	v	g	m
CS:1	0.013539	0.06342	0.03016	0.05329
Cer d18:1/23:0	0.023397	0.331	0.3715	0.40043
DG 18:0_20:4	0.010394	0.08773	0.15158	0.17385
DG 18:2_18:2	0.029179	11.8225	8.148	9.61857
Hex2Cer d18:1/16:0	0.031583	0.10025	0.0757	0.09114
Hex-Cer d18:2/24:0	0.043678	0.2795	0.2172	0.24107
Ind-SO4	0.045327	15.4942	13.331	15.9043
LPE 19:0	0.04476	0.07883	0.1071	0.10379
LPG 14:1	0.014855	0.031	0.03871	0.04515
MG 18:1	0.041919	284.075	167.84	186.893
MG 20:3	0.013288	5.54928	1.62326	2.56101
PC 40:2	0.043462	0.42075	0.4515	0.48086
PC O-32:2	0.047386	0.269	0.3005	0.314
PC O-36:2	0.023927	12.8617	14.57	15.0714
PC O-38:2	0.0064713	9.27917	11.934	12.1464
PC O-38:3	0.040974	2.14	2.315	2.39929
PC O-40:4	0.035938	2.1625	2.284	2.53143
PE 33:2	0.043175	0.41258	0.4686	0.48807
PE 35:2	0.016853	2.59333	2.866	3.05429
PG 16:0_19:1	0.03316	0.02852	0.02746	0.04515
PG 16:0_20:3	0.024823	0.18183	0.2361	0.20986
PI 16:0_16:0	0.027223	0.42767	0.5535	0.55157
PS 36:3	0.030505	0.09575	0.0861	0.09721
SM 34:1	0.04308	18.5417	18.36	19.7571
SM 42:1	0.024369	5.9225	6.182	6.72643
SPBP d18:0	0.037045	0.41558	0.2746	0.33271
TG 17:0_36:3	0.042067	1.5375	1.00984	1.59707
TG 17:2_36:3	0.022002	0.70458	0.42996	0.53679
TG 17:2_38:6	0.040349	0.28907	0.15324	0.23396
TG 18:1_35:3	0.043267	1.87167	1.3443	1.92
TG 18:1_36:3	0.047028	158.858	107.16	138.871
TG 18:1_38:6	0.033549	7.8275	5.586	7.05571
TG 18:2_36:0	0.04165	1.96245	1.70214	2.58714
TG 18:2_38:4	0.030261	5.92417	4.275	5.72429
TG 20:3_36:3	0.025796	5.79583	4.139	5.03929
TG 20:3_36:4	0.042704	1.96417	1.3784	1.81071
TG 20:4_35:3	0.031818	0.09732	0.06298	0.10457
TG 22:4_32:2	0.034299	0.29783	0.21042	0.25157
TG 22:6_34:2	0.039429	17.2175	11.403	16.0686

B.

9 weeks	ANOVA_p	v	g	m	v-g	v-m	m-g
C16:1	1.43123666[illegible]	0.05708	0.0648	0.08621	0.14337	2.31396E-05	0.0009
Lac	2.03674879[illegible]	11931	12617.7	19463.8	0.61608	5.23146E-05	0.00076
PE P-20:0/20:5	0.0002162	0.15042	0.1657	0.25871	0.44463	0.000411334	0.00377
Suc	0.0002356	23.2417	30.71	52.5857	0.0131	0.000882627	0.00813
C2	0.0002958	10.9917	13.78	15.7821	0.03104	2.78967E-05	0.13067
C6 (C4:1-DC)	0.0003266	0.05675	0.0752	0.08929	0.01621	0.000159261	0.077
PE P-18:1/20:5	0.000352	0.06563	0.0835	0.12523	0.15028	6.59512E-05	0.01122
C14:1	0.0003915	0.0555	0.067	0.07979	0.06198	0.000141136	0.0407
CE 20:5	0.0004047	31.7083	33.44	57.6857	0.74642	0.000814022	0.00263
PE P-16:0/20:5	0.0004365	0.11658	0.1365	0.23507	0.4249	0.000496052	0.00944
PC 34:3	0.0008461	16.015	16.15	20.9071	0.92392	0.002163557	0.00106
PE P-18:0/20:5	0.0008476	0.27383	0.3234	0.49036	0.32145	0.000524572	0.01652
beta-Ala	0.0008852	3.305	2.741	4.55357	0.12504	0.010121313	0.00091
SM 36:2	0.0009129	0.77075	0.7794	0.9845	0.86637	0.001890148	0.00223
CE 16:1	0.0014748	31.5917	36.53	54.0429	0.30147	0.001489617	0.0124
PC 36:5	0.0014859	7.9575	7.58	10.2943	0.6304	0.007739802	0.00044
LPC 16:1	0.0015986	9.52417	11	13.9357	0.10239	0.001672943	0.01835
PE P-18:1/22:6	0.0016674	2.33917	2.5	3.03643	0.34765	0.000891926	0.02377
C16-OH	0.0019786	0.001	0.001	0.00143	1	0.008088745	0.00809
Asn Synthesis	0.0019823	2.39583	3.302	2.08779	0.01004	0.297843055	0.00193
CE 18:3	0.0021936	25.0833	25.14	36.6143	0.98609	0.002746824	0.00607
Hex-Cer d18:1/20:0	0.0022774	0.64925	0.7237	0.93321	0.4097	0.001967549	0.01195
CE 14:0	0.0024105	3.74	4.14	5.51	0.38068	0.001414095	0.01982
PC 36:3	0.0027366	147.508	144.8	186	0.8064	0.008325779	0.00167
LPE 20:3	0.0027759	1.01158	1.0374	1.31529	0.74414	0.003313942	0.00784
CE 20:3	0.0027903	17.375	19.41	29.1357	0.45854	0.002569167	0.01535
Hexose	0.0028644	5864.58	7644.5	7004.86	0.00026	0.019826393	0.20261
PC O-38:0	0.0028656	3.07167	3.125	4.02714	0.86125	0.008262219	0.00076
Hex2Cer d18:1/22:0	0.0031299	0.07242	0.0617	0.09336	0.23789	0.017691026	0.00271
PE P-16:0/16:0	0.0032475	0.16975	0.1861	0.25914	0.46426	0.002822873	0.01482
SM 36:1	0.0033871	1.46	1.656	1.96357	0.21339	0.000381076	0.09003
Taurine	0.0036022	431.667	406.5	465.214	0.10228	0.056088856	0.00071
Glu	0.0037039	76	85.33	125.057	0.46317	0.002195426	0.02691
Cer d18:1/24:1	0.0038576	1.45667	1.47	1.88786	0.91369	0.005993098	0.00543
SM 34:2	0.0038971	3.00667	2.988	3.77857	0.9389	0.007817278	0.00314
PG 18:1_20:3	0.0041886	1.006	1.0591	1.28929	0.57946	0.00289402	0.0152
alpha-AAA	0.0042818	7.30917	6.94	9.89286	0.68319	0.013487702	0.00159
PG 18:2_20:5	0.0046379	0.35258	0.3735	0.46814	0.47693	0.003586951	0.0211
C18:2	0.004662	0.05942	0.0717	0.08471	0.10778	0.000952969	0.13315
PE P-18:1/18:1	0.0047011	0.583	0.735	0.883	0.02938	0.002505281	0.14575
p-Cresol-SO4	0.0048811	20.2291	7.4042	20.8029	0.00505	0.897748042	0.0006
PI 16:0_20:3	0.0049038	1.00883	1.0845	1.31614	0.38402	0.003831266	0.0186
p-Cresol-SO4 Synthesis	0.0050551	0.17767	0.0558	0.18214	0.00447	0.917417743	0.00052
PC O-30:2	0.0052124	0.05258	0.0606	0.07086	0.10558	0.00212569	0.08608
PE 38:0	0.0052251	0.86417	0.8361	1.043	0.63128	0.008296607	0.00904
CE 22:6	0.0054284	180.083	179.72	245.286	0.98574	0.005005277	0.01374
PC O-44:5	0.0056997	0.49008	0.5209	0.66979	0.61594	0.006020393	0.00963
CE 16:0	0.0057086	36.5333	41.74	57.6857	0.32815	0.003486493	0.03695
LPC 26:0	0.0061931	0.24142	0.268	0.30979	0.25317	0.001185951	0.08603
PG 18:1_20:4	0.0063438	3.175	3.332	4.06857	0.613	0.005532062	0.01353
LPC 16:0	0.0064252	348.583	367.4	448.857	0.44481	0.005426894	0.02447
C16	0.0065669	0.1625	0.1866	0.23029	0.27543	0.001045129	0.09107
C18:1	0.0066889	0.164	0.1902	0.23571	0.17468	0.002589692	0.08058
Carnosine	0.0066917	0.91642	1.0031	1.38579	0.47906	0.004291861	0.03448
LPC 26:1	0.0068228	0.21058	0.234	0.26579	0.14931	0.003341985	0.07053
CE 18:2	0.0068993	611.833	663.6	928.214	0.49786	0.006191195	0.02425
PC 32:3	0.0071078	0.17233	0.1842	0.21529	0.46327	0.002501225	0.04506
CE 15:0	0.0071468	1.90133	1.901	2.963	0.99922	0.005622974	0.01904
PC 34:1	0.0072765	146.208	150.4	192.143	0.79878	0.009594161	0.00674
Hex-Cer d18:1/24:1	0.0078179	8.5775	9.047	11.5086	0.58427	0.004930281	0.02777
LPC 18:1	0.0080259	131.55	147.8	175.429	0.1776	0.005431686	0.04737
Hex2Cer d18:1/24:1	0.0083609	0.05883	0.0619	0.07457	0.64544	0.001014843	0.06014
PC O-28:0	0.0085271	0.48933	0.5239	0.67679	0.57846	0.003266169	0.03876
Cer d18:2/24:1	0.0089309	0.12733	0.1121	0.14786	0.16556	0.0933824	0.0007
LPE 15:0	0.0091586	0.19682	0.2029	0.28693	0.78163	0.011807906	0.01332
PC 36:6	0.0094254	0.50158	0.4797	0.60436	0.60393	0.032297217	0.00137
PE P-18:0/22:6	0.0097968	11.0058	11.1	13.6429	0.92307	0.003963722	0.03272
PC 32:2	0.0099324	1.32792	1.3502	1.64143	0.84976	0.00782028	0.01732
PC 34:4	0.0100795	0.76017	0.7455	0.92357	0.81488	0.026179207	0.00179
PC 42:0	0.0102921	0.26892	0.2893	0.34764	0.51001	0.001588308	0.07111
Anserine	0.0104951	1.72342	1.8302	3.16286	0.73928	0.013342829	0.02048
PG 18:1_20:5	0.0108625	0.32917	0.3512	0.42621	0.48617	0.007536541	0.02763
PE P-18:0/22:2	0.0110822	1.24658	0.9669	0.81479	0.09165	0.007624342	0.23333
PE 40:1	0.0111893	0.12983	0.1324	0.17293	0.84761	0.009882237	0.02122
PG 18:1_20:0	0.0117769	0.2625	0.3132	0.3505	0.08366	0.00383576	0.22482
PG 18:1_22:4	0.011794	0.90542	0.9633	1.14129	0.47821	0.006320657	0.03805
PI 15:0_16:0	0.012572	5.5475	5.669	6.94143	0.8048	0.012745499	0.01442
PE 44:12	0.0125905	0.19	0.1874	0.239	0.87474	0.014032214	0.01731
LPC 20:3	0.0126394	20.025	23.62	28.2357	0.14215	0.006208644	0.10019
PI 16:0_17:2	0.0129997	8.9375	8.969	10.8029	0.96663	0.018313599	0.0076
PI 18:0_20:3	0.0131431	3.53	3.959	4.67643	0.34039	0.004546413	0.08522
PG 18:2_20:0	0.0131845	0.40342	0.455	0.51364	0.07713	0.007120635	0.12466
PE P-18:1/18:2	0.0133947	0.87067	1.3211	1.496	0.04147	0.002994058	0.49156
AconAcid	0.0139858	3.00917	4.44	9.50714	0.01622	0.016105082	0.05262
LPS 18:1	0.0140893	0.28075	0.342	0.3885	0.02893	0.007391244	0.22034
CE 17:1	0.0141796	1.76417	1.841	2.80071	0.76661	0.013639262	0.03325
PC O-32:2	0.0142768	0.24742	0.2898	0.34186	0.1765	0.003214629	0.16342
ProBetaine	0.0144096	1.78583	1.905	2.37357	0.32322	0.014079521	0.03871
PC O-44:6	0.0144698	0.90708	0.9463	1.11136	0.62419	0.005672006	0.04633
PC 38:3	0.0145897	32.8417	33.96	43.1714	0.75125	0.012995179	0.0202
PE P-18:0/18:1	0.0148373	0.83717	0.8524	1.08621	0.85292	0.00911736	0.03793
Cer d18:2/24:0	0.0151446	0.05842	0.0705	0.0825	0.15823	0.005982423	0.14991
PE P-16:0/15:0	0.0153095	1.05692	1.1294	1.52607	0.61334	0.011181681	0.03316
PG 16:0_20:5	0.0153421	0.26483	0.2742	0.32671	0.68781	0.014821373	0.01624
PC O-32:1	0.0156208	0.56542	0.6497	0.76571	0.26196	0.001678546	0.17743
OH-GlutAcid	0.0160142	3.72333	4.416	8.55714	0.02303	0.020958811	0.04309
PC 34:2	0.0164999	425.25	417.2	504.286	0.79992	0.022096103	0.01272
PC 40:3	0.0165115	0.63883	0.6674	0.85407	0.66935	0.011553898	0.03605
CE 22:5	0.0166104	4.3475	5.118	6.50571	0.23273	0.007154108	0.09457
Cer d18:1/22:0	0.0176876	0.72983	0.7688	0.95236	0.6701	0.006203289	0.05353
PC O-42:2	0.0186742	0.44283	0.4695	0.57021	0.55073	0.019227946	0.01739
LPE P-18:1	0.0186984	0.29533	0.2309	0.32657	0.03672	0.375104592	0.00175
Hex-Cer d18:1/22:0	0.0188904	4.18667	4.477	5.58857	0.59793	0.009273404	0.04684
LPC 24:0	0.0189433	0.66892	0.7137	0.843	0.39438	0.0096205	0.06755
Thr	0.0191375	214.083	203.1	244.643	0.36279	0.049395509	0.01052
LPE 20:1	0.0192018	0.4055	0.4123	0.50071	0.83548	0.015618728	0.02829
PI 17:1_18:1	0.019422	3.99583	4.073	4.78286	0.82538	0.015619143	0.02487
PG 17:1_18:1	0.0195631	0.32842	0.3615	0.30071	0.09511	0.186289344	0.00505
Val	0.0197668	133.833	202.9	174.071	0.04677	0.004774762	0.35725
PE 32:1	0.020742	0.03367	0.0332	0.04871	0.92953	0.030852575	0.01146
PE 32:2	0.0210744	0.079	0.0753	0.09557	0.574	0.045329089	0.00815
PC O-36:0	0.021656	0.4295	0.4579	0.53607	0.50631	0.005700495	0.08733
PC 32:1	0.0233501	5.7325	5.877	7.39786	0.84603	0.02682314	0.01323
PC O-40:2	0.0238094	0.63533	0.6841	0.80321	0.37643	0.011487406	0.07804
PC 42:4	0.0244858	0.21092	0.221	0.26207	0.57401	0.012664143	0.05829
LPE 16:0	0.0246912	4.96167	4.934	5.96143	0.91629	0.032578438	0.02576
Gln	0.025218	728.75	877.4	930.929	0.07218	0.004534828	0.54697
PC O-38:6	0.0254634	2.13083	2.354	2.82	0.37042	0.006499555	0.1285
PE P-16:0/22:6	0.0258776	6.9725	7.202	8.90929	0.77636	0.005780126	0.08704
PC 40:2	0.0259205	0.35925	0.3844	0.46721	0.51638	0.010260914	0.07936
PG 18:2_20:4	0.0263712	10.6683	10.536	12.4914	0.8661	0.023874107	0.02733
PE 38:1	0.0264276	0.25742	0.2789	0.33757	0.40645	0.011006743	0.09581
PG 18:2_20:3	0.0264689	2.94417	2.949	3.48643	0.98127	0.02387109	0.02818
PC O-40:1	0.0265417	4.31667	4.075	5.23286	0.59127	0.055055283	0.00811
CE 20:4	0.0270564	757.833	765.1	982.929	0.93028	0.020674478	0.03691
PC O-42:3	0.0272324	1.35325	1.2808	1.71429	0.67821	0.050564691	0.00833
PC 36:1	0.0273296	39.9167	43.1	52.3143	0.54546	0.008510932	0.08722
LPC 18:2	0.0275485	359.167	392.9	451.071	0.29878	0.009947502	0.12342
PC 38:6	0.0276972	118.6	115.88	142.571	0.81799	0.042229078	0.01038
PG 16:0_20:3	0.0281058	0.20458	0.2172	0.25879	0.56217	0.016742974	0.05068
PS 36:4	0.0285118	0.369	0.3926	0.54136	0.63238	0.023291546	0.04618
PS 36:1	0.0293931	0.0655	0.0817	0.12329	0.21185	0.019110153	0.09892
PG 18:1_22:5	0.0297471	1.14958	1.2086	1.42571	0.42306	0.023494719	0.05578
Asp	0.0302447	30.1333	30.66	43.3	0.93053	0.009663862	0.06656
SPB d18:1	0.0309724	0.2035	0.19656	0.32783	0.89029	0.038357872	0.02046
PE 20:0	0.0314847	0.11283	0.1101	0.13929	0.80686	0.055462064	0.00737
PE 36:0	0.0316546	0.54075	0.567	0.67879	0.65973	0.005951761	0.10846
PE P-18:1/20:4	0.0318501	1.55917	1.821	2.085	0.20555	0.004992317	0.27822
PE 38:7	0.0320483	0.30733	0.2957	0.39814	0.72632	0.042365543	0.0238
Ind-SO4	0.0321287	16.8408	16.84	26.1714	0.99962	0.041041961	0.03828
PC O-42:4	0.032764	0.34483	0.3648	0.42314	0.50765	0.019062783	0.06179
PC O-34:1	0.0332466	5.05583	5.383	6.31571	0.49271	0.013072814	0.09328
TG 20:5_36:2	0.0332686	2.29083	2.0724	2.99929	0.49422	0.064367492	0.01488
LPE 22:6	0.0335326	7.5875	7.783	9.06929	0.77784	0.018454597	0.05152
Hex-Cer d18:2/24:0	0.0345509	0.22733	0.2523	0.29886	0.27746	0.016243335	0.11987
PE P-18:0/18:3	0.0349403	0.16792	0.1745	0.21993	0.77371	0.007318063	0.10518
PC O-34:0	0.0352534	0.52742	0.5526	0.66836	0.62158	0.019706544	0.06585
MA (NBS)	0.0357156	0.06333	0.0457	0.05207	0.00955	0.073323209	0.3571
PG 16:0_22:1	0.0357184	0.90133	0.9012	1.02464	0.99802	0.026364598	0.03956
Serotonin	0.0370158	32.7667	47.41	41.1929	0.00578	0.127620443	0.22921
PC O-38:3	0.0384998	1.98667	2.188	2.57143	0.3624	0.014853337	0.12691
LPE P-17:0	0.0385765	0.09727	0.072	0.09823	0.02973	0.927795436	0.01698
PC 36:4	0.0389382	175.417	171.5	203.5	0.79301	0.067702936	0.00646
CE 18:1	0.0395168	118.775	133.56	176.071	0.46205	0.015214149	0.1191
Cer d18:2/16:0	0.0399419	0.03942	0.0402	0.05129	0.8692	0.030608411	0.04225
PG 18:1_22:3	0.040965	0.32967	0.332	0.40157	0.9452	0.024840052	0.05046
PC 38:1	0.0410026	0.28258	0.3001	0.37179	0.63903	0.018912642	0.07796
Hex-Cer d18:1/18:0	0.041211	0.42183	0.4527	0.51321	0.46536	0.022954993	0.08663
SM 42:1	0.0412595	5.6275	6.28	7.85643	0.41664	0.014120031	0.13786
PC O-36:3	0.0417131	2.43083	2.756	3.19214	0.28731	0.007996318	0.23344
PC O-42:5	0.042233	0.688	0.7576	0.82557	0.22534	0.008367819	0.28283
PE P-16:0/18:1	0.0423302	0.78983	0.8108	1.02757	0.83081	0.018123184	0.08199
CE 18:0	0.0425992	3.73833	4.373	5.48929	0.30932	0.013115281	0.17951
LPE 18:0	0.0429743	5.06917	5.109	5.92643	0.89033	0.030712443	0.06374
PC 38:0	0.0436947	1.785	2.041	2.36643	0.31651	0.005981944	0.25227
SM 42:2	0.0438126	12.65	13.06	15.9214	0.73022	0.021942434	0.08697
Hex-Cer d18:2/23:0	0.0452023	0.13467	0.20714	0.17	0.05943	0.045142462	0.30714
PC O-36:1	0.045403	2.65833	2.94	3.37714	0.33399	0.0136773	0.17688
FA 20:5n-3 (EPA)	0.0467623	2.82083	2.575	3.34571	0.45115	0.098230829	0.01735
PC 42:1	0.047241	0.154	0.1716	0.1995	0.41705	0.003485217	0.24785
Choline	0.0479127	66.8083	63.32	81.15	0.7012	0.013916709	0.08027
PE 34:4	0.0482599	0.03358	0.0276	0.041	0.21823	0.194661457	0.00751
PE P-18:0/20:4	0.0488315	8.46	8.56	10.3693	0.91535	0.012121702	0.10422
PS 38:6	0.0493709	0.4525	0.4621	0.63921	0.84724	0.048266724	0.05223

C.

10 day post	ANOVA_p	v	g	m	v-g	v-m	m-g
PA 18:1_18:1	0.0001338	0.8381	0.59238	0.98267	8.5414E-05	0.075732631	0.000119922
PA 18:0_18:1	0.0002474	1.511	1.1242	1.84357	0.023349661	0.057057226	8.62187E-06
PA 18:1_18:3	0.0030766	1.6725	1.04656	1.70592	0.006676194	0.873202058	0.000941063
PA 18:1_20:3	0.0150312	3.07167	2.721	3.805	0.358018932	0.059434229	0.003868405
PA 18:2_18:2	0.022559	1.19875	0.8099	1.41957	0.066622779	0.344073904	0.001192232
LPE 14:0	0.0275733	0.06622	0.14925	0.09358	0.051735667	0.090716634	0.169386058
PA 18:1_20:1	0.028651	0.55144	0.48022	0.62015	0.202398577	0.191231667	0.009638594
MG 16:1	0.0309427	11.4942	6.766	9.87214	0.00819314	0.364563934	0.036317047
PA 18:2_22:4	0.0309528	1.72225	1.767	2.25929	0.840767231	0.029243453	0.023334734
PA 16:2_18:1	0.031091	5.25417	4.33	6.07214	0.158257664	0.223292561	0.003848387
PA 18:1_22:3	0.0484325	1.54525	1.4435	1.92786	0.614753932	0.074825361	0.019563712

D

Muscle	ANOVA_p	v	g	m	v-g	v-m	m-g
PG 18:2_20:5	5.387E-05	0.70842	0.8732	0.63464	0.003587085	0.0885924	0.0001392
PE 40:5	9.374E-05	252.333	353.7	248.857	0.000924481	0.8657496	0.0005833
PE 40:4	9.512E-05	33.0083	44.71	33.3	0.001981227	0.8783551	0.0027509
PE 38:4	0.0003182	273	383.9	276.643	0.004600347	0.8473553	0.0062361
PG 18:0_18:2	0.0003251	1.69333	2.384	1.73929	0.000336441	0.772276	0.0007249
PE 44:7	0.000329	1.96833	2.846	2.32429	7.7237E-05	0.0546391	0.0143426
PA 18:2_20:2	0.0004129	57.4333	96.86	58.4286	0.001376622	0.9096274	0.0016502
PG 16:1_20:4	0.0004527	9.93333	13.651	9.54429	0.006363574	0.6106295	0.0036986
PE 34:4	0.0004555	1.24583	1.659	1.33	0.003274121	0.2469728	0.0123565
PE 38:5	0.0010074	221.5	265.3	230.286	0.003222047	0.3603281	0.0084598
PE 44:6	0.0012921	0.31067	0.5304	0.3775	0.00151112	0.1403399	0.0269873
PC O-40:4	0.001371	4.4025	5.973	4.80143	0.002358186	0.2471682	0.0141709
PE 42:8	0.0016211	15.575	19.18	16.6857	0.001478799	0.1908804	0.0143901
Cer d18:1/18:1	0.0016986	0.34317	0.3838	0.46029	0.206361359	0.0003534	0.0545204
Gln	0.0017747	1091.5	1440.3	1111.93	0.00443404	0.8033733	0.0073511
PG 16:0_18:2	0.0018518	12.7083	16.7	14.3643	0.002550723	0.0959142	0.0253637
PG 16:0_16:0	0.0020884	2.185	2.826	2.98571	0.006243227	0.0017298	0.4617184
PI 18:0_18:2	0.0021298	15.3317	22.37	16.7143	0.00287202	0.4158226	0.0099551
TG 22:5_34:1	0.0026253	2.63583	4.22	3.85143	0.004662862	0.0033251	0.4548365
PG 18:1_18:2	0.0032392	14.325	17.9	15.9429	0.003529953	0.0595328	0.0701395
Cer d18:1/26:0	0.0033319	0.2341	0.32267	0.41177	0.04749542	0.0008967	0.1181222
PC 40:4	0.0040731	14.1358	18.67	14.2743	0.009152052	0.9163808	0.0045704
PA 18:2_22:3	0.0049587	8.34583	11.28	9.15643	0.00879908	0.247127	0.0366546
PI 18:2_20:1	0.0054066	1.15333	1.372	1.17179	0.010197042	0.7231155	0.0239558
TG 22:6_34:1	0.0058161	5.04333	7.702	7.96357	0.018683501	0.0013518	0.8111514
PA 18:2_22:4	0.0058815	20.4833	28.36	21.3143	0.004474158	0.7213334	0.0073282
PS 38:7	0.0060216	0.87358	0.9541	1.07429	0.191629678	0.0028917	0.0575334
PI 18:2_22:6	0.0074242	1.15825	1.438	1.23471	0.011379812	0.2285995	0.0591531
PC O-36:2	0.0074593	16.6167	20.67	18.0143	0.004821026	0.2163227	0.0366732
PG 18:2_22:1	0.0080461	1.34225	1.731	1.2745	0.047982875	0.5867127	0.0118695
PG 18:2_18:2	0.0080601	8.4625	11.668	9.01286	0.014627544	0.4615861	0.0424517
TG 16:0_40:6	0.0085861	1.94333	2.887	2.48929	0.01840306	0.0086762	0.2728012
PI 18:0_20:4	0.0086879	283	336.5	313.429	0.006909853	0.086124	0.0742438
HCys	0.0088878	14.8558	8.767	11.5564	0.003313673	0.1029511	0.0580046
PG 16:0_18:1	0.009074	65.2833	82.11	72.3357	0.009413067	0.1429013	0.0628227
C4	0.009543	2.7375	3.885	2.65286	0.043453581	0.7694027	0.0294908
PC O-38:4	0.0099787	15.3083	18.47	16.1571	0.006854722	0.38166	0.0180125
PS 40:5	0.0100052	85.9583	104.37	89.7429	0.009321914	0.4912247	0.0189331
Cer d18:1/23:0	0.0102654	2.46667	3.524	4.16857	0.045077908	0.0029726	0.302946
PC 38:4	0.0103056	251.75	305.1	258.857	0.010750213	0.6815135	0.007798
PE 40:8	0.0104988	250.333	301.3	266.214	0.013905317	0.2673423	0.044694
PE 36:4	0.0105613	73.85	93.44	76.0643	0.036255389	0.5752282	0.0578222
PI 16:0_18:2	0.0108537	4.15	5.159	4.40643	0.012776916	0.3459579	0.0439051
PI 18:0_22:0	0.0110954	1.58	1.868	1.49214	0.056337007	0.3960881	0.0122313
Asp	0.0117671	31.325	51.49	33.6929	0.008859321	0.6942703	0.0220335
PG 20:4_22:4	0.0123807	0.49208	0.5602	0.42079	0.209878798	0.0598279	0.0149395
PE 42:7	0.012516	24.6417	32.66	27.5786	0.016100768	0.1593409	0.0891905
Cer d18:1/24:0	0.0125781	8.1975	12.347	16.7121	0.128916967	0.0030278	0.1825745
TG 22:5_32:0	0.0130077	0.78125	1.2442	1.22621	0.025471316	0.0074388	0.9199872
TG 22:5_34:2	0.0130824	3.21417	4.712	4.15714	0.004041297	0.0350786	0.3016241
PI 18:1_18:2	0.0132683	5.77	7.2	6.15786	0.01268088	0.3232124	0.0537627
Cer d18:1/22:0	0.0135266	3.29	4.82	5.77714	0.071607556	0.0029012	0.3218483
PE 38:3	0.0137411	14.6	18	15.7643	0.018570121	0.1436928	0.1087615
PI 16:0_18:1	0.0137458	8.3875	10.341	9.26429	0.01634656	0.0790205	0.1470265
TG 17:2_36:2	0.0143732	2.18667	1.584	2.33	0.026423896	0.6071594	0.0004606
PG 16:1_18:2	0.0144507	14.4358	19.01	14.1643	0.051603366	0.8155792	0.0406496
PC 42:5	0.0148559	1.14458	1.4846	1.21036	0.014990077	0.4869316	0.0458152
TG 22:6_32:0	0.0148676	2.314	4.037	3.93714	0.0088466	0.010144	0.8849471
TG 14:0_38:5	0.0152049	0.59233	0.9194	1.06633	0.029494354	0.0087846	0.4488869
PI 18:1_20:3	0.0154786	2.48167	2.39	2.845	0.592699051	0.0367602	0.0053621
Hex-Cer d18:1/26:0	0.0163546	0.80458	1.2885	1.6965	0.141958729	0.0025308	0.2570289
PC 32:0	0.0164923	213.583	249.2	256.5	0.054595858	0.0073121	0.6430024
PA 18:2_20:1	0.0169076	25.2417	33.05	27.1714	0.025859064	0.3429222	0.0713147
C16:2	0.0169573	0.85567	1.4141	0.98843	0.022468181	0.3979815	0.0557631
PE 40:6	0.017976	1221.33	1494.1	1306.64	0.011190036	0.3073399	0.0553225
Betaine	0.0198119	49.1333	64.56	54.5357	0.026271799	0.1384142	0.1362733
PE P-16:0/22:4	0.0199328	56.7083	72.64	60.15	0.034615568	0.3380061	0.0957263
PC O-36:4	0.0199996	11.2892	13.468	12.0979	0.013658987	0.2224299	0.0833867
Hex-Cer d18:1/24:0	0.0200005	43.4167	68.81	92.4714	0.15529474	0.0035789	0.246889
TG 22:4_34:2	0.0202643	1.36625	2.1	1.7855	0.004134213	0.086786	0.2052151
PE P-18:0/18:3	0.0206328	0.57158	0.824	0.62807	0.034735922	0.3246483	0.0988352
Hex-Cer d18:1/23:0	0.0208629	19.3917	29.253	38.0786	0.149592371	0.003675	0.2619258
PC 40:5	0.0210581	59.025	75.67	56.0714	0.056458769	0.6534086	0.0124468
PE P-20:0/16:0	0.0213036	1.75333	2.287	2.06643	0.008186431	0.0754429	0.2323373
PG 18:0_18:3	0.0214831	1.30333	1.496	1.25293	0.01660978	0.5301717	0.0150309
PG 17:0_18:1	0.0222188	0.50425	0.5998	0.63364	0.12660434	0.0011418	0.5660089
PC O-38:2	0.0223809	5.15667	6.537	5.23143	0.026411552	0.8699614	0.0293373
PI 16:1_18:1	0.0226562	8.49417	9.364	9.47	0.2926456	0.128728	0.8764136
PC 34:3	0.0227482	98.175	116.73	106.836	0.020358803	0.1758624	0.0884416
PI 18:2_20:0	0.0249983	5.17833	6.363	5.34786	0.02666478	0.6652388	0.0361533
TG 16:0_40:7	0.0252228	2.18692	3.131	3.05429	0.040551857	0.0141487	0.8465254
PC O-36:3	0.0270236	7.96583	10.172	9.06571	0.017942474	0.1306839	0.1610278
PG 18:0_22:1	0.0270836	0.54908	0.6513	0.48071	0.198057434	0.1956607	0.022625
C3:1	0.0275848	0.15373	0.20638	0.1657	0.046480398	0.3785096	0.1054786
PC O-42:5	0.0284231	4.14833	5.187	4.38429	0.034215845	0.4521105	0.072138
LPE 14:1	0.028884	0.76883	1.413	1.01521	0.026559586	0.1732931	0.1474465
PA 18:1_18:2	0.03248	3.76917	4.872	4.00857	0.034102118	0.4309225	0.1010427
PI 17:1_18:2	0.0328566	19.2833	24.77	17.835	0.105024586	0.4547343	0.0454225
TG 20:4_32:0	0.0331304	1.80383	3.004	2.8305	0.010608953	0.0270284	0.7412433
PI 18:0_20:0	0.0348327	18.3792	24.31	18.4786	0.041850339	0.9650101	0.026231
PG 18:0_18:1	0.0350262	2.49333	2.953	2.81214	0.022613195	0.0605405	0.4136311
C14	0.0351721	1.44608	2.232	1.75286	0.028496135	0.2235996	0.1298474
PC O-34:1	0.0353083	46.875	51.92	54.5429	0.165235614	0.0132539	0.3840026
C6 (C4:1-DC)	0.0353754	0.69267	0.9072	0.71093	0.048739328	0.8129452	0.037691
Asn	0.0356253	25.9917	34.12	25.95	0.051100025	0.9869604	0.0616863
PI 18:1_22:5	0.0356429	2.49917	2.634	3.085	0.649884405	0.0047681	0.1276881
C12	0.0357598	0.62592	0.8628	0.77057	0.028644493	0.078954	0.3096502
PI 18:2_22:0	0.0358266	0.57542	0.6612	0.54571	0.122884576	0.3814766	0.040795
PE 32:2	0.0362019	2.19167	2.868	2.45143	0.037611782	0.1376467	0.1919531
PC 42:0	0.0370383	0.56975	0.7035	0.553	0.080004332	0.7433801	0.0350223
PS 40:8	0.0375869	4.5375	5.303	5.21571	0.042104323	0.0263125	0.7895913
Cit	0.0376227	59.0417	71.85	72.9071	0.064917313	0.029379	0.8402953
Hex2Cer d18:1/20:0	0.0393576	0.48142	0.69211	0.79071	0.103248856	0.0092631	0.506546
Cer d18:1/20:0	0.0414793	1.50017	2.0866	2.38429	0.126134355	0.0079464	0.4662362
PC 42:6	0.0415851	3.84667	5.098	3.955	0.052855631	0.8026517	0.0562438
Hex2Cer d18:1/22:0	0.0419989	0.15827	0.22838	0.20431	0.020726571	0.0602121	0.4124061
TG 22:6_32:1	0.0420403	2.79358	3.707	4.69857	0.138203708	0.0156994	0.2401652
PE 30:1	0.0424006	0.233	0.2979	0.25107	0.045251061	0.2995201	0.1471967
Hex-Cer d18:1/22:0	0.0433562	41.65	62.91	81.4071	0.203535039	0.008125	0.3177001
TG 22:6_34:2	0.043594	5.19083	7.183	7.46	0.015677126	0.025661	0.7820501
PE 38:2	0.044245	6.57333	7.806	7.03857	0.025976341	0.2555006	0.1413832
PI 18:2_20:4	0.0458396	1.44833	1.739	1.45357	0.077826893	0.9480592	0.0953248
C16:1	0.0472297	1.73825	2.746	2.22	0.034589944	0.1391326	0.2311287
TG 17:2_38:6	0.0474447	0.9053	0.661	1.18827	0.237775504	0.2208635	0.0059035
LPE 22:5	0.0481114	3.03917	3.749	3.08857	0.025096339	0.8534907	0.0540759
C18:2	0.0483778	3.26175	5.522	4.06357	0.041514834	0.2548875	0.158973

E

Brain	ANOVA_p	v	g	m	v-g	v-m	m-g
MG.22.1	0.0100113	11.8542	12.691	17.4164	0.67472	0.00838	0.01701
TG.22.4_34.2	0.0111717	0.56078	0.30763	0.42508	0.01087	0.07099	0.14009
alpha.AAA	0.0267551	18.4042	12.227	14.1514	0.00978	0.0731	0.32496
PG.16.0_22.2	0.0280545	1.56833	1.2966	1.707	0.06895	0.34185	0.00858
C3.1	0.0303051	0.21142	0.19638	0.16786	0.45156	0.01638	0.10103
PE.P.18.1.20.5	0.034499	10.2833	8.87	10.9214	0.06468	0.42506	0.0052
Cer.d18.2.24.0	0.0503652	4.42833	3.159	4.13857	0.0415	0.5127	0.0866

F

Liver	p.value	v	g	m	v-g	v-m	m-g
Betaine	0.012547	294.83333	382.1	378.5	0.15976	0.00456	0.95022
PG 22:4_22:6	0.013088	0.3186667	0.3409	0.27729	0.43812	0.0197	0.02837
Hex-Cer d18:2/24:0	0.014168	0.3376667	0.28075	0.28271	0.05798	0.03379	0.93979
PC O-42:0	0.016783	3.0633333	3.422	3.44	0.02358	0.00786	0.89894
PC O-38:1	0.02004	0.9486667	1.219	1.08621	0.01835	0.05996	0.19873
PC 28:1	0.022222	0.2449167	0.3434	0.28529	0.00785	0.24701	0.11948
DG 16:1_18:2	0.026438	12.038333	16.598	14.2321	0.02411	0.13799	0.2436
1-Met-His	0.031455	0.5804167	0.5672	0.64843	0.85246	0.34184	0.04554
Ala	0.038139	938.75	802.3	914.357	0.01949	0.68793	0.03747
PA 18:2_20:0	0.039563	0.9154167	1.0534	0.72179	0.47279	0.12475	0.09204
PI 18:0_20:3	0.040487	26.708333	26.44	30.2214	0.87353	0.02213	0.0281
Hex-Cer d18:1/22:0	0.044242	15.458333	13.18	17.2214	0.22397	0.10354	0.05118
PI 17:0_18:1	0.048176	0.49425	0.6281	0.50729	0.04181	0.81445	0.06795
PE 42:7	0.049507	4.0008333	4.614	4.69786	0.06072	0.01167	0.78215

Table S3.

Mixed-effects longitudinal analysis of metabolite concentration in serum collected at baseline, at the 9th week of treatment (T1) and 10 days after washout (T2) in mice receiving vehicle, GinA (10 mg/kg) (g) or modGinA (27 mg/kg) (m) daily for 10 weeks.

var	(Intercept)	tpT1	tpT2	g2_g	g3_m	tpT1:g2_g	tpT2:g2_g	tpT1:g3_m	tpT2:g3_m	pval	padj	model	null
alpha-AAA	7.836666667	-0.5275	6.004166667	-0.805666667	0.176904762	0.4365	-1.766166667	2.406785714	-2.520595238	0.013701931	0.374547199	N	N
Anserine	1.969166667	-0.24575	-0.222333333	-0.209166667	-0.010595238	0.31595	-0.103066667	1.450035714	0.066261905	0.025012674	0.443695211	Y	Y
beta-Ala	3.289166667	0.015833333	0.69	0.525833333	0.389404762	-1.089833333	0.043	0.859166667	0.044285714	0.016940908	0.402064222	N	N
CE 14:0	3.804166667	-0.064166667	2.314166667	-0.222166667	0.298690476	0.622166667	0.817833333	1.471309524	-0.926309524	0.033908139	0.443695211	N	Y
CE 15:0	1.952727273	0.055454545	1.494772727	-0.085227273	0.325606061	0.315795455	0.413727273	0.783135198	-0.908106061	0.020812375	0.443695211	Y	Y
CE 16:0	32.56666667	3.966666667	23.16666667	-0.466666667	4.99047619	5.673333333	6.703333333	16.16190476	-12.4952381	0.011190711	0.331991097	N	Y
CE 16:1	42.325	-10.73333333	29.89166667	0.565	3.039285714	4.373333333	5.808333333	19.41190476	-9.341666667	0.047791383	0.459830601	N	N
CE 18:1	76.8	41.975	101.775	-9.04	-0.542857143	23.825	27.465	57.83928571	-29.44642857	0.03714623	0.443695211	N	N
CE 18:2	581.5	30.33333333	345.75	10	81	41.76666667	83.95	235.3809524	-173.3214286	0.009112549	0.29734704	N	N
CE 18:3	24.75	0.333333333	12.09166667	-0.76	2.128571429	0.816666667	2.988333333	9.402380952	-5.913095238	0.009187689	0.29734704	N	N
CE 20:3	20.70833333	-3.333333333	5.875	-2.411333333	0.548809524	4.446333333	3.268	11.21190476	-1.682142857	0.028795963	0.443695211	N	N
CE 20:4	691.9166667	65.91666667	133.1666667	-71.11666667	28.58333333	78.38333333	86.13333333	196.5119048	-105.0238095	0.045491222	0.443695211	N	N
CE 20:5	35.65833333	-3.95	22.50833333	-1.558333333	4.170238095	3.29	-2.908333333	21.80714286	-9.222619048	0.003722601	0.199057331	N	N
Cer d18:1/24:0	0.5705	-0.06675	0.231416667	-0.0009	0.070285714	0.10875	-0.052816667	0.064964286	-0.230059524	0.027486751	0.443695211	N	N
Cer d18:1/24:1	1.358166667	0.0985	0.499333333	-0.180766667	-0.129452381	0.1941	0.081266667	0.560642857	0.018380952	0.034131224	0.443695211	Y	Y
Cer d18:2/24:0	0.076	-0.017583333	0.02525	-0.0086	0.005357143	0.020683333	-0.00495	0.01872619	-0.023321429	0.004020605	0.199057331	N	N
Choline	46.10833333	20.7	5.275	-1.648333333	-2.544047619	-1.84	3.905	16.88571429	5.989285714	0.041087577	0.443695211	N	N
GABA	0.520416667	-0.054416667	0.0925	0.230683333	0.019154762	-0.144083333	0.1305	0.439488095	0.0885	0.030639994	0.443695211	N	N
Hex-Cer d18:1/20:0	0.84525	-0.196	-0.212083333	-0.18745	-0.117035714	0.2619	0.170883333	0.401	0.16622619	0.003001941	0.183272733	N	N
Hex-Cer d18:1/24:1	7.43	1.1475	3.649166667	-0.623	-0.36	1.0925	0.166833333	3.291071429	-0.531309524	0.044074546	0.443695211	Y	Y
Lac	13596	-1665	5310	-826.3	123.0714286	1513	261.3	7409.714286	-2567.571429	1.21E-05	0.008609005	Y	Y
LPE 18:0	5.4775	-0.408333333	1.900833333	-0.3995	0.056071429	0.439333333	-0.041833333	0.801190476	-1.011547619	0.045442543	0.443695211	N	Y
LPE P-18:1	0.20575	0.089583333	0.078666667	-0.01095	0.050122615	-0.053483333	0.019133333	-0.018884519	-0.113303655	0.006705623	0.265244661	N	N
MG 18:1	284.075	-175.125	-70.09166667	-116.235	-97.18214286	109.645	47.22166667	112.375	72.04880952	0.044435919	0.443695211	N	N
MG 20:3	6.046187167	-3.735296772	-2.5545205	-3.36949941	-2.78919703	3.075505694	1.833999414	2.481323277	2.771958935	0.042190769	0.443695211	N	Y
PA 16:2_18:1	6.608333333	2.310833333	-1.354166667	-0.174333333	-0.461190476	1.039166667	-0.749833333	0.13202381	1.279166667	0.008147738	0.290059473	N	N
PA 17:1_18:1	1.39	0.194166667	-0.03520515	0.319	0.062857143	-0.102166667	-0.61859485	-0.099166667	0.010062293	0.014729384	0.374547199	N	N
PA 17:2_18:1	1.8575	0.1725	-0.176068355	0.0725	-0.233928571	0.2965	-0.480483385	0.135357143	0.379639784	0.024820846	0.443695211	N	N
PA 18:0_18:1	1.17475	0.751916667	0.33625	0.08035	0.002107143	-0.174016667	-0.46715	-0.196630952	0.330464286	0.00050669	0.072152654	N	N
PA 18:1_18:1	0.993666667	-0.029929051	-0.161262442	-0.011066667	-0.06102381	0.141229051	-0.22842291	0.043071908	0.202413461	0.002856994	0.183272733	N	N
PA 18:1_18:3	1.660583333	-0.003916667	0.001571079	0.055416667	-0.028154762	0.185916667	-0.666565247	0.168916667	0.0586024	0.03887843	0.443695211	N	N
PA 18:1_18:4	0.93	0.097583333	-0.181067282	-0.0732	0.029214286	0.22936476	0.121265244	-0.06322619	0.170352996	0.033324497	0.443695211	N	N
PA 18:1_20:3	4.395	1.115833333	-1.323333333	-0.084	-0.448571429	0.804166667	-0.266666667	-0.04297619	1.181904762	0.004778981	0.200154977	N	N
PA 18:1_22:3	1.540833333	1.165	0.004416667	0.080166667	-0.154404762	0.345	-0.181916667	0.048571429	0.537011905	0.003088866	0.183272733	N	N
PA 18:2_18:2	0.827416667	0.16725	0.371333333	-0.012816667	0.149654762	0.27345	-0.376033333	-0.246892857	0.071166667	0.02728258	0.443695211	N	N
PA 18:2_22:3	1.454166667	0.808333333	0.032513164	0.067733333	0.129404762	0.346766667	-0.243313164	-0.237619048	-0.070370307	0.022456656	0.443695211	N	N
PC O-34:0	0.455166667	0.07225	0.20175	0.019633333	0.064833333	0.00555	-0.00595	0.076107143	-0.138321429	0.032750527	0.443695211	N	N
PC O-36:0	0.363083333	0.066416667	0.148083333	0.047316667	0.041988095	-0.018916667	-0.037483333	0.064583333	-0.094154762	0.040655185	0.443695211	N	N
p-Cresol-SO4	0.642274789	19.58680854	18.81005854	-0.275125236	-0.21510578	-12.5497581	-7.663308097	0.78887959	-4.860084696	0.037292176	0.443695211	N	N
PE 33:1	0.214166667	0.090416667	0.050583333	0.017833333	0.023047619	0.010883333	0.013616667	0.068940476	-0.029940476	0.034958196	0.443695211	N	N
PE P-16:0/15:0	0.919083333	0.137833333	0.392	-0.124383333	-0.065011905	0.196866667	0.3205	0.534166667	0.104214286	0.032380068	0.443695211	Y	Y
PE P-16:0/16:0	0.206083333	-0.036333333	-0.00325	0.006416667	-0.018083333	0.009933333	0.03405	0.10747619	0.043634615	0.004473198	0.199057331	Y	Y
PE P-16:0/18:1	0.466333333	0.3235	0.053416667	-0.012233333	0.024595238	0.0332	0.112883333	0.213142857	0.036083333	0.041997085	0.443695211	N	N
PE P-16:0/20:5	0.163166667	-0.046583333	0.0195	-0.006166667	-0.003238095	0.033400684	-0.0106	0.12172619	0.002785714	0.000199944	0.053294903	N	Y
PE P-18:0/18:1	0.35775	0.479416667	0.220916667	0.04205	0.02525	-0.026816667	0.025083333	0.223797619	0.022797619	0.016417777	0.402064222	N	N
PE P-18:0/20:5	0.279166667	-0.005333333	0.118416667	-0.018466667	-0.006595238	0.068033333	-0.004716667	0.223119048	0.031869048	0.00098599	0.117004203	N	Y
PE P-18:1/18:1	0.488333333	0.094666667	-0.046416667	-0.013433333	0.038809524	0.165433333	0.148316667	0.261190476	0.06327381	0.010002849	0.30965342	N	N
PE P-18:1/18:2	2.008916667	-1.13825	-0.658	8.33E-05	0.196797619	0.45035	0.8654	0.428535714	0.231285714	0.036656921	0.443695211	N	N
PE P-18:1/20:4	1.7675	-0.208333333	-0.066666667	-0.0535	-0.040357143	0.315333333	0.514666667	0.566190476	0.195952381	0.03117625	0.443695211	N	N
PE P-20:0/20:5	0.153272727	-0.002856061	0.048143939	0.008027273	-0.01962987	0.007256061	0.000156061	0.127927489	0.024213203	0.000387762	0.069021695	Y	Y
PG 16:0_16:0	0.671666667	0.124833333	-0.17425	0.027933333	-0.033809524	0.088966667	-0.09875	-0.040833333	0.089678571	0.027635301	0.443695211	N	N
PG 16:0_18:1	0.791416667	0.207166667	-0.172083333	-0.007916667	-0.091416667	0.303533333	-0.066716667	0.089047619	0.213011905	0.007999633	0.290059473	N	N
PG 16:0_20:3	0.181833333	0.02275	0.039416667	0.054266667	0.02802381	-0.04165	-0.060316667	0.026178571	-0.015916667	0.040391664	0.443695211	N	N
PG 16:2_18:1	0.22375	0.063166667	0.0095	-0.01585	-0.000892857	0.005233333	-0.0083	-0.017738095	0.072357143	0.023528242	0.443695211	N	N
PG 17:0_18:1	0.110333333	0.063439557	0.115835462	0.007766667	-0.011261905	0.02050124	-0.040670084	0.027830486	0.016164032	0.037481594	0.443695211	N	Y
PG 17:1_18:1	0.225166667	0.10325	0.012	-0.010066667	-0.018166667	0.04315	0.0047	-0.009535714	0.054285714	0.000224557	0.053294903	N	N
PG 18:1_18:1	0.552833333	0.226416667	0.085333333	-0.055933333	-0.09697619	0.216283333	0.131566667	0.119940476	0.215166667	0.002535265	0.183272733	N	N
PG 18:1_20:0	0.266083333	-0.003583333	-0.001333333	-0.038383333	-0.009297619	0.089083333	0.038333333	0.097297619	0.039333333	0.02870987	0.443695211	N	N
PG 18:1_20:1	0.266666667	0.030416667	-0.044159121	0.011733333	-0.021595238	0.027983333	-0.001153518	0.078511905	0.068301979	0.043455767	0.443695211	N	N
PG 18:2_20:0	0.44225	-0.038833333	0.028916667	-0.01295	0.032535714	0.064533333	0.009883333	0.077690476	-0.026202381	0.037707058	0.443695211	N	N
PI 16:0_16:0	0.427666667	0.084833333	0.159017531	0.125833333	0.123904762	-0.108833333	-0.220717531	-0.098119048	-0.128231816	0.044448874	0.443695211	N	Y
PI 18:1_20:1	0.249583333	0.142716667	0.078527778	0.023861111	-0.033869048	0.012172222	-0.117372222	-0.045645238	0.065357937	0.043470624	0.443695211	Y	Y
PI 18:1_20:2	0.482416667	0.149083333	-0.047809079	-0.009516667	-0.067702381	0.120316667	-0.080492742	0.022559524	0.140951936	0.013035078	0.371239035	N	N
PI 18:1_22:0	0.2095	-0.046488031	-0.00494643	-0.010062417	-0.020214286	0.045510237	0.007943184	0.084636147	0.006192404	0.032423237	0.443695211	N	N
PS 36:1	0.055333333	0.010166667	0.02025	0.001166667	-0.007315337	0.015033333	0.02555	0.065101051	0.006303432	0.02195017	0.443695211	N	N
SM 34:1	18.54166667	2.275	9.508333333	-0.181666667	1.21547619	3.275	1.821666667	5.117857143	-4.21547619	0.039665598	0.443695211	N	N
SM 35:1	0.225166667	0.033083333	0.11275	-0.008366667	0.021404762	0.053116667	0.03145	0.04877381	-0.066535714	0.028325507	0.443695211	N	N
SM 36:1	1.259166667	0.200833333	0.5725	-0.077166667	0.011547619	0.273166667	0.0895	0.49202381	-0.280714286	0.001445932	0.147071949	N	N
SM 36:2	0.620916667	0.149833333	0.258583333	-0.061516667	0.022154762	0.070166667	0.059916667	0.191595238	-0.132583333	0.004347717	0.199057331	N	Y
SM 42:1	5.9225	-0.295	3	0.2595	0.803928571	0.393	0.214	1.425	-1.669285714	0.039012671	0.443695211	N	N
SM 42:2	10.07916667	2.570833333	7.820833333	-0.606166667	0.755119048	1.016166667	0.166166667	2.516309524	-3.472261905	0.03832682	0.443695211	N	N
SM 43:1	0.107583333	0.07725	0.175916667	0.021216667	0.034916667	0.00705	-0.029016667	0.027464286	-0.099916667	0.002749169	0.183272733	N	N
TG 18:2_36:0	2.099245014	-0.857411681	0.765754986	-0.237437226	0.487897843	1.517967237	1.266437226	0.231406248	-0.9100407	0.04409033	0.443695211	N	N
Thr	258.6666667	-44.58333333	-0.583333333	-22.56666667	-20.45238095	11.58333333	52.48333333	51.01190476	6.011904762	0.014546405	0.374547199	Y	Y

Footnote:

var=metabolited

(Intercept) = intercept of mixed-effects model

tpT1 = coefficient associated with timepoint T1

tpT2 = coefficient associated with timepoint T2

g2_g = coefficient associated with group GinA

g3_m = coefficient associated with group modGinA

tptb:g2_g = coefficient associated with timepoint T1 / group GinA interaction

tpT2:g2_g = coefficient associated with timepoint T2 / group GinA interaction

tpT1:g3_m = coefficient associated with timepoint T1 / group modGinA interaction

tpT2:g3_m = coefficient associated with timepoint T2 / group modGinA interaction

pval = unadjusted p-value

padj = false discovery rate (FDR) adjusted p-value

model = model was singular (N=No, Y=Yes)

null = null model was singular (N=No, Y=Yes)

Table S5. The ratio of relative TdTomato staining over DAPI between groups receiving vehicle (high oleic acid sunflower oil), 10 mg/kg GinA or 27 mg/kg of modGinA daily for 10 days prior to and 20 days after i.p. administration of 10 mg/kg doxorubicin (vehicle) or without doxorubicin administration (negative control) in different tissues (one-way ANOVA). Log base 10 scale on relative units . See Figure 3B for scatterplots.

	Lung	Brain	Liver	Kidney
**Negative Control**	0.003646973	0.003558719	0.003471407	0.00039748
**Negative Control**	0.006510915	0.000638978	0.007423858	0.001073537
**Negative Control**	0.000872981	0.001716738	0.00423357	3.7221E-05
**Negative Control**	0.006820119	0.00171969	0.005795235	0.000608167
**Negative Control**	0.002902758	0.001629992	0.00238691	0.000600996
**Vehicle**	0.013560501	0.000724113	0.028754496	0.000399848
**Vehicle**	0.170660147	0.001663894	0.003794832	0.001988072
**Vehicle**	0.148689516	0.003873824	0.00591937	0.002980626
**Vehicle**	0.133333333	0.004872107	0.024716562	0.001614205
**Vehicle**	0.021310182	0.007616975	0.007064452	0.002610966
**Vehicle**	0.012401353	0.001943005	0.006200281	0.004382121
**Vehicle**	0.013367609	0.00074239	0.004552998	0.007675439
**GinA**	0.002918856	0.00014627	0.009649291	0.000799041
**GinA**	0.004733728	0.000788731	0.010087685	0.000833824
**GinA**	0.004968383	0.000848896	0.000458817	0.004590164
**GinA**	0.008248423	0.007735584	0.010186148	0.000378136
**GinA**	0.011996161	0.007027407	0.008795344	0.000636821
**GinA**	0.013116057	0.002846975	0.002514669	0.001055188
**GinA**	0.019358125	0.00065189	0.004226957	0.002333722
**GinA**	0.020661157	0.004204625	0.004239125	0.001609658
**modGinA**	0.005952381	0.000617251	0.009003556	0.003382187
**modGinA**	0.006659836	0.000387597	0.004774114	0.001770695
**modGinA**	0.007944915	0.001302083	0.015190111	0.000256576
**modGinA**	0.004001455	0.002449479	0.00569444	0.000922509
**modGinA**	0.010117361	0.005514706	0.020979021	0.000931966
**modGinA**	0.003665988	0.000857633	0.001983362	0.001716002
**modGinA**	0.020862968	0.002046385	0.007474788	0.000446403
**modGinA**	0.002842255	0.000802568	0.004992903	0.00010731

Table S6. Precision of NAD+ metabolites that were measured

	% CV
NAD	3.28
NADH	9.4
NMN	9.44
NADP	6.26
NADPH	14.91
NAM	7.69
NAMN	5.16
AcCoA	4.16
ADPR	5.5
NAAD	3.42
